# Analysis of Wheat Wax Regulation Mechanism by Liposome and Transcriptome

**DOI:** 10.3389/fgene.2021.757920

**Published:** 2021-12-06

**Authors:** Hongwei Wen, Ying Wang, Bangbang Wu, Yanru Feng, Yifei Dang, Bin Yang, Xiaofei Ma, Ling Qiao

**Affiliations:** ^1^ State Key Laboratory of Integrative Sustainable Dryland Agriculture, Institute of Wheat Research, Shanxi Agricultural University, Linfen, China; ^2^ Crop Science, Institute of Crop Science and Resource Conservation (INRES), University of Bonn, Bonn, Germany

**Keywords:** wheat, wax, liposome, transcriptome, molecular regulation

## Abstract

As a barrier for plants to contact with the outside world, epidermal wax plays an important role in resisting biotic and abiotic stresses. In this study, we analyzed the effect of wax content on leaf permeability by measuring the wax loss rate in the leaf. To further clarify the wax composition of the wheat epidermis and its molecular regulation mechanism, we applied untargeted lipidomic and transcriptome analysis on the leaf epidermis wax of Jimai 22 low-wax mutant (*waxless*) and multi-wax mutant (*waxy*). Our research showed that the mutant *waxy* has a slow loss rate, which can maintain higher leaf water content. 31 lipid subclasses and 1,367 lipid molecules were identified. By analyzing the wax differences of the two mutants, we found that the main lipid components of leaf epidermis wax in Jimai 22 were WE (C19-C50), DG (C27-C53), MG (C31-C35), and OAHFA (C31-C52). Carbon chain length analysis showed that, in wheat epidermis wax, WE was dominated by C44 molecules, DG was mainly concentrated in C47, C45, C37, and C31 molecules, C48 played a leading role in OAHFA, and C35 and C31 played a major role in MG. Among them, DG, MG, and OAHFA were detected in wheat leaf wax for the first time, and they were closely related to stress resistance. Compared with the *waxy*, 6,840 DEGs were detected in the mutant *waxless*, 3,181 DEGs were upregulated, and 3,659 DEGs were downregulated. The metabolic pattern of main waxy components in the wheat epidermis was constructed according to KEGG metabolic pathway and 46 related genes were screened, including *KSC*, *TER*, *FAR*, *WSD1*, *CER1*, *MAH1*, *ALDH7A1*, *CYP704B1*, *ACOT1_2_4*, *CYP86*, *MGLL*, *GPAT*, *ALDH*, *DPP1*, *dgkA*, *plsC,* and *E2.3.1.158* related genes. The screened wax-related genes were confirmed to be highly reliable by qRT-PCR. In addition, we found *TER* gene *TraesCS6B03G1132900LC* in wheat mutant *waxless* leaves for the first time, which inhibited the synthesis of long-chain acyl-CoA (n+2) by downregulating its expression. These results provide valuable reference information for further study of wheat epidermis wax heredity and molecular regulation.

## Introduction

As the first barrier for plants, epidermis wax plays an important role in resisting biotic and abiotic stresses (Aharoni et al., 2004; Franke et al., 2005; Bernard and Joubes, 2013). For example, it can limit the loss of non-stomatal water, improve the drought resistance of plants, help plants reduce mechanical damage, plant diseases, and insect pests, and protect plants from high temperature and strong ultraviolet radiation (Reina-Pinto and Yephremov, 2009; Yeats et al., 2012). Epidermal wax is a complex mixture of lipids composed of very-long-chain fatty acids (VLCFAs) and their derivatives (Zhang et al., 2005; Tafolla-Arellano et al., 2018). Existing studies have shown that the biosynthesis of epidermal wax begins with a waxy forerunner transformed by very-long-chain fatty acids C16 or C18 on the outer membrane of plastid epidermal cells. The carbon chains of C16 or C18 acyl-CoA and malonyl-CoA are lengthened by β-ketoacyl-CoA synthetase (KCS), β-ketoacyl-CoA reductase (KCR), β-hydroxyacyl-CoA dehydratase (HCD), and enoloyl-CoA reductase (ECR). Then, these very-long-chain fatty acids synthesize different waxy compounds by acyl reduction and decarbonylation (Samuels et al., 2008; Dong et al., 2019). Because of the complexity of waxy biosynthesis, the accurate determination of plant waxy components and content is helpful to infer the pathway of plant waxy biosynthesis accurately. However, most of the existing studies on epidermal wax components are based on gas chromatography-mass spectrometry (GC-MS), and some trace components cannot be detected, which leads to some limits in the study of the epidermal wax synthesis pathway.

Many genes involved in the wax synthesis and regulation have been found in *Arabidopsis* and rice (Racovita et al., 2016; Shaheenuzzamn et al., 2019). For instance, transcription factor *WIN1/SHN1* in *Arabidopsis* upregulates the expression of epidermal wax synthesis genes *CER1*, *CER2*, *CER4*, *KCS*, *CYP86A7*, *CYP86A4*, *GPAT4*, *LACS2,* and *HTH* to induce epidermal wax accumulation (Kannangara et al., 2007). The overexpression of *CER1* leads to the accumulation of alkanes (Bourdenx et al., 2011), and overexpression of *CER4* (*AtFAR3*) induces the production of primary alcohols in C24:0-C30:0 (Rowland et al., 2006). Zhou et al. (2014) found that *OsWR2* in rice, as a homologue of *AtWIN1/AtSHN1*TF, controls wax synthesis and accumulation by regulating the expression of very-long-chain fatty acid biosynthesis genes *CER6/CUT1*, *FDH2*, *FAE,* and *LACS1* in the panicle. Due to the huge genomic information of wheat, there are few studies on the molecular regulation mechanism of wheat epidermal wax biosynthesis. It has been reported that *W1*-*W5*, *Iw1*, *Iw2,* and *Iw3* are related to the wax synthesis of the wheat epidermis (Huang et al., 2017; Li et al., 2020). Li et al. (2021) studied the wax-deficient mutant *w5* and found that the blockage of β-diketone biosynthesis inhibited waxy synthesis. Chai et al. (2018) cloned several *TaFARs* genes encoding fatty acyl-CoA reductase from wheat.

In view of the complexity of wheat epidermis wax composition and synthesis mechanism, the analysis of wheat wax deletion mutants is considered to be a tool to get many response genes. To study the regulation mechanism of wheat epidermis wax, low-wax mutants and multi-wax mutants obtained by ethyl methanesulfonate (EMS) mutagenesis of Jimai 22 were used as materials in this study. By means of mutual verification and joint analysis of untargeted liposome and transcriptome, the main components of wheat epidermis wax were identified, and new genes related to epidermal wax metabolism were excavated, such as *TraesCS1D03G0373900*, *TraesCS1D03G0374000*, *TraesCS4B03G0019500*, *TraesCS7B03G1338900*, *TraesCS5B03G0557800*, *TraesCS5D03G0511400*, and *TraesCS7A03G0874000*, which improved the possible molecular regulation mechanism of wheat epidermis wax synthesis. The purpose of this study is to provide valuable reference information for further study on the genetic and molecular mechanism of epidermis wax metabolism in wheat, which provides theoretical support for wheat breeding and genetic improvement.

## Materials and Methods

### Plant Materials

**FIGURE 1:**
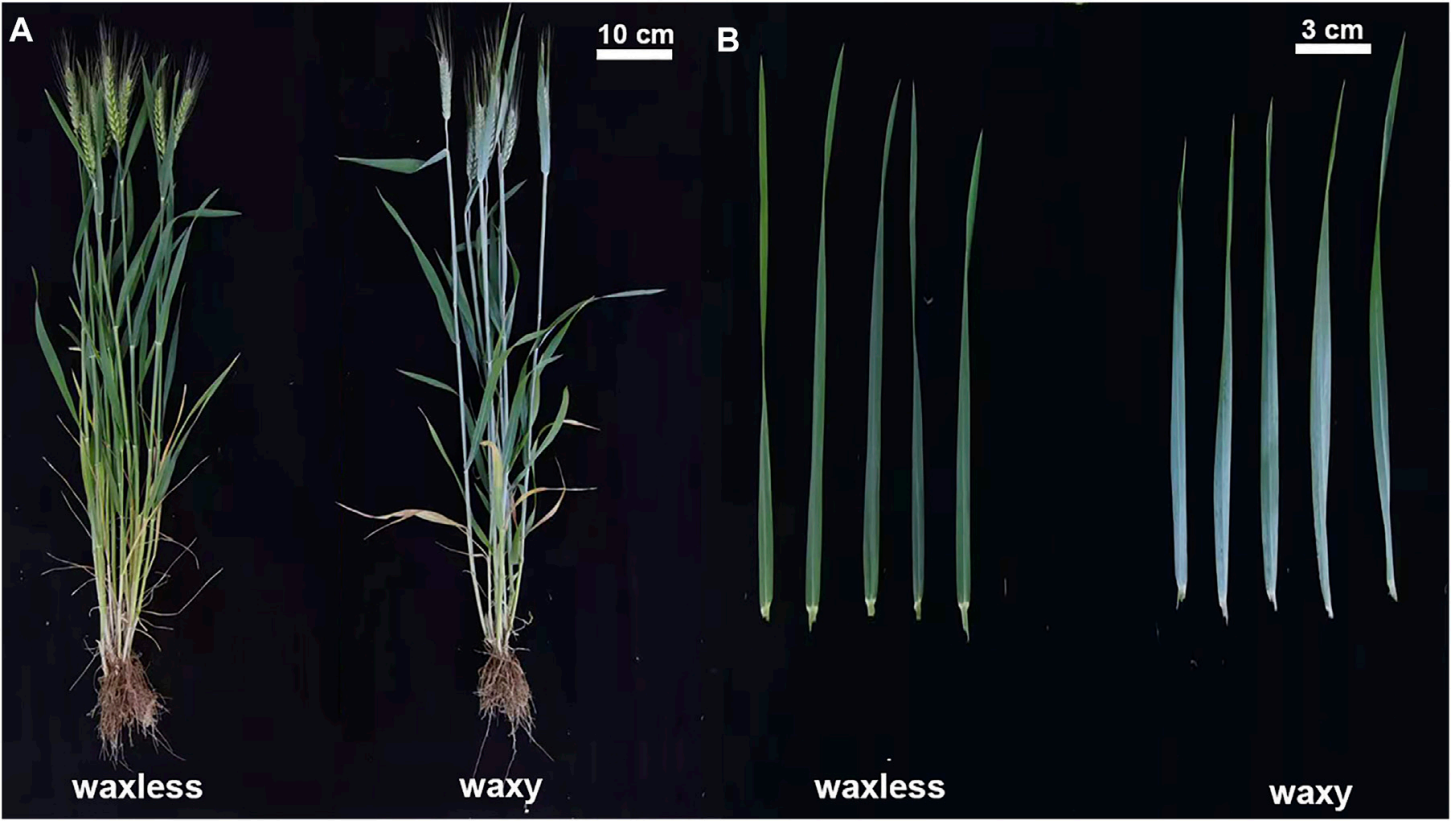
Phenotype of low-wax mutant *waxless* and multi-wax mutant *waxy*. **(A)** The whole plant phenotype of *waxless* and *waxy*. **(B)** Leaf phenotype of *waxless* and *waxy*.

Jimai 22 is a high-yielding variety selected by the Crop Research Institute of Shandong Academy of Agricultural Sciences, containing wax in wild leaf epidermis. Combined with the experimental experience of our research group for many years, 0.6% (v/w) EMS was used to mutagenize Jimai 22 in this study, and then *waxless* and *waxy* were selected from the separated high-generation population. Compared with *waxy*, *waxless* showed less wax in the whole plant (Figures 1A,B). In October 2020, the mutants were planted in the Hancun Experimental Base of Wheat Research Institute of Shanxi Agricultural University (36°N, 111°E), with three replicates. The length was 2 m, the row spacing was 0.3 m, and 30 seeds per row were sown evenly. Wax content and transcriptome analysis were performed on flag leaves at the heading stage. Three biological repeats were performed for both *waxless* and *waxy*, and two samples with good repeatability were selected in transcriptome analysis.

### Determination of Water Loss Rate of Leaves

In order to analyze the effect of leaf epidermis wax on the water loss rate of wheat leaves, in the same period as transcriptome sequencing, the flag leaves at the heading stage of *waxy* and *waxless* mutants were fully soaked and dehydrated in the dark at room temperature for 10 h (stomata were completely closed). The leaf water loss rate was calculated by 0.001 mg analysis balance (AUW320, Japan) every hour. Each sample was repeated three times.

### Untargeted Lipidomics Detection

Liposome was detected by liquid chromatogramphy-tandem mass spectrometry (LC-MS). The leaves were immersed in chloroform for 30 s to dissolve the epidermis wax and dried with nitrogen. Lipids were extracted according to the MTBE method (Pizarro et al., 2013). Briefly, samples were spiked with a suitable amount of internal lipid standards and then homogenized with 200 µl water and 240 µl methanol. After that, 800 µl of MTBE was added and the mixture was ultrasound-sonicated 20 min at 4°C followed by keeping still for 30 min at room temperature. The solution was centrifuged at 14,000 g for 15 min at 10°C and the upper organic solvent layer was obtained and dried under nitrogen. Reverse-phase chromatography was selected for LC separation using the CSH C18 column (1.7 µm, 2.1 × 100 mm, Waters). The lipid extracts were re-dissolved in 200 µl 90% isopropanol/acetonitrile, centrifuged at 14,000 g for 15 min; finally, 3 µl of the sample was injected. Solvent A was acetonitrile-water (6:4, v/v) with 0.1% formic acid and 0.1 Mm ammonium formate and solvent B was acetonitrile-isopropanol (1:9, v/v) with 0.1% formic acid and 0.1 Mm ammonium formate. The initial mobile phase was 30% solvent B at a flow rate of 300 μl/min. It was held for 2 min and then linearly increased to 100% solvent B in 23 min, followed by equilibrating at 5% solvent B for 10 min. Mass spectra were acquired by Q-Exactive Plus in positive and negative modes, respectively. ESI parameters were optimized and preset for all measurements as follows: source temperature was set at 300°C, capillary temp at 350°C, the ion spray voltage at 3000 V, S-Lens RF level at 50% and the scan range of the instruments at m/z 200–1800.

### Extraction of RNA and Transcriptome Sequencing

The clean leaves of the two mutants were taken at the heading stage, wrapped in tin foil, frozen in liquid nitrogen quickly, and then frozen in the refrigerator at −80°C. The samples were sent to Beijing Baimaike Biotechnology Co., Ltd., for transcriptome sequencing. Total RNA was extracted using the Trizol method, and the concentration and purity of RNA were measured by NanoDrop 2000 (Thermo Fisher Science, Wilmington, DE). RNA integrity was evaluated using the RNA Nano 6000 analysis kit of Agilent Biological Analyzer 2,100 system (Agilent Technologies, CA, United States ). A total amount of 1 μg RNA per sample was used as input material for the RNA sample preparations. Sequencing libraries were generated using NEBNext Ultra TM RNA Library Prep Kit for Illumina (NEB, United States ) following the manufacturer’s recommendations and index codes were added to attribute sequences to each sample. In order to select cDNA fragments of preferentially 240 bp in length, the library fragments were purified with the AMPure XP system (Beckman Coulter, Beverly, United States ). Then, 3 μl USER Enzyme (NEB, United States ) was used with size-selected, adaptor-ligated cDNA at 37°C for 15 min followed by 5 min at 95°C before PCR. PCR was performed with Phusion High-Fidelity DNA polymerase, Universal PCR primers, and Index (X) Primer. At last, PCR products were purified (AMPure XP system) and library quality was assessed on the Agilent Bioanalyzer 2,100 system.

### Real-Time Quantitative PCR Analysis

Primers were designed by Primer Premier Version 5.0 (Premier Biosoft International, Palo Alto, CA) and combined by Sangon (www.sangon.com) (Supplementary Table S1). LA-Taq enzyme from TaKaRa (www.takara.com.cn) was used for PCR amplification. PCR was performed in total volumes of 15 μl, including 3 pmol of each primer, 120 μM of each dNTP, 80 ng template DNA or cDNA, 0.75 unit La-Taq, and 7.5 μl of 2 × buffer (TaKaRa Biotechnology (Dalian) Co., Ltd., Product Code: DRR20AG). PCR was performed as follows: 95°C for 4 min, followed by 35 cycles of 95°C for 30 s, annealing (55–62°C) for 30 s, extension at 72°C (30 s–3 min), and 72°C for 30 s, with a final extension of 72°C for 10 min. Annealing temperatures and extension times depended upon individual primer sets and the length of expected PCR products.

Quantitative real-time PCR was performed using SYBR® Premix Ex Taq™ II (Takara) according to the manufacturer’s instructions on a 7,300 Real-time PCR System (Applied Biosystems), where the relative expression of each gene was calculated according to the 2^−ΔΔCT^ method (Zheng et al., 2020). The glyceraldehyde-3-phosphate dehydrogenase gene was used as an endogenous reference for real-time PCR, and all analyses were performed with three technical and three biological replications.

### Data Analysis

“Lipid Search” is a search engine for the identification of lipid species based on MS/MS math. Lipid Search contains more than 30 lipid classes and more than 1,500,000 fragment ions in the database. Both mass tolerances for precursor and fragment were set to 5 ppm. Under the positive and negative ion modes, the OPLS-DA model was constructed by SAMIC14.1 software, and the prediction rate Q^2^ of the model was obtained by 7-fold cross-validation. Q^2^ > 0.5 was taken as the reliable standard of the model; the multiple of variation analysis (Fold Change Analysis, FC) > 4 or < 0.25 and the variable importance for the projection (VIP) > 1, *p* value < 0.05, were used as the screening criteria to compare the overall differential expression multiples of lipid ions in leaves of *waxless* and *waxy*. Lipid differences were analyzed with SPSS19.0 and Microsoft Excel 2019, plotted with Origin 2018. The transcriptome data were further processed by the online platform of BMK Cloud (www.Biocloud.net). FPKM (Fragments Per Kilobase of exon model per Million mapped fragments) method was used to standardize the gene expression level. DESeq R package was used for differential analysis, and Fold Change ≥2 and FDR < 0.01 were used as screening criteria to determine the differentially expressed genes (DEGs) between *waxless* and *waxy* mutants.

## Results and Analysis

### Effect of Epidermis Wax on Water Loss Rate of Wheat Leaves

**FIGURE 2:**
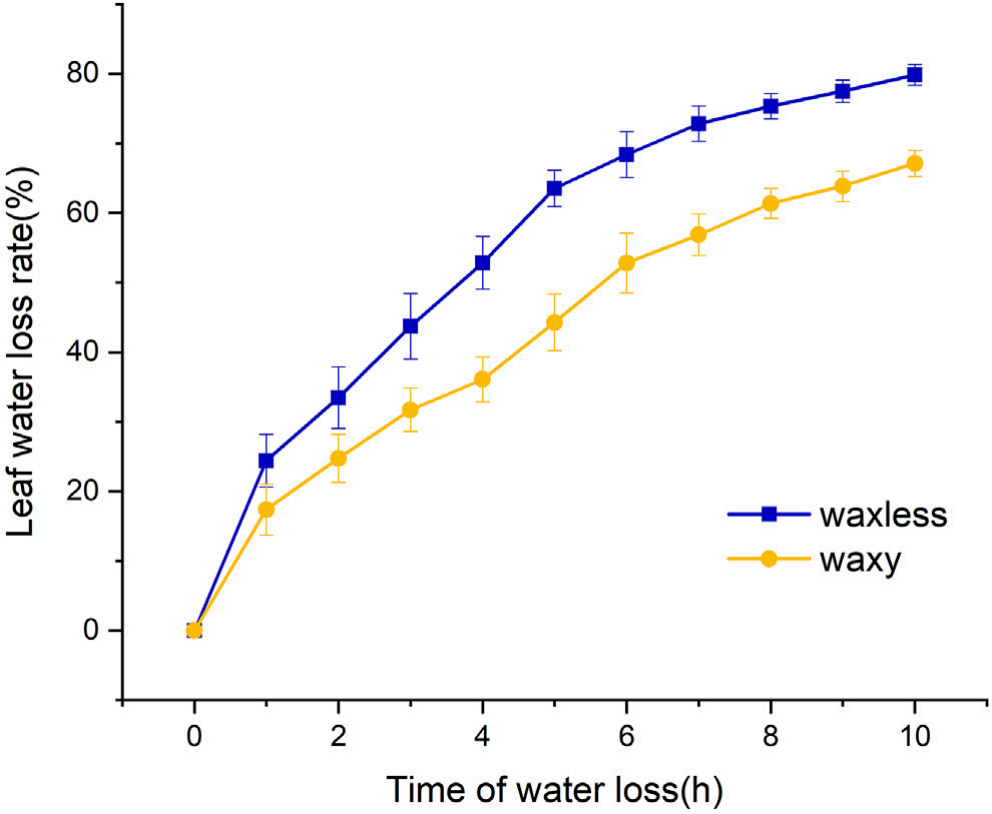
Water loss rate of flag leaves detached from mutant *waxless* and mutant *waxy*.

The leaf water loss rate of the two mutants was faster in the darkroom temperature environment. The wax content had a significant effect on the leaf dehydration rate. After natural dehydration for 1 h, the water loss rate of low-wax mutant leaves reached 24.4% and that of multi-wax mutant leaves reached 17.4%. The leaves of the two mutants were dehydrated rapidly within 1–5 h, and the water loss rate of *waxless* was significantly higher than that of *waxy*. After dehydration for 5 h, the water loss rate of *waxless* leaves reached 63.5%, while that of *waxy* was only 44.3%. There was a significant difference between the two mutants. Although the dehydration rate of the two mutants slowed down after 5 h of dehydration, the water loss rate of low-wax mutants was still significantly higher than that of wax-rich mutants at 10 h of dehydration (Figure 2). This shows that the mutant *waxy* has a slow dehydration rate and strong water retention capacity and can maintain higher leaf water content, indicating that leaf wax content plays an important role in maintaining leaf water content.

### Identification and Analysis of Lipid Components in Leaf Epidermis

**FIGURE 3:**
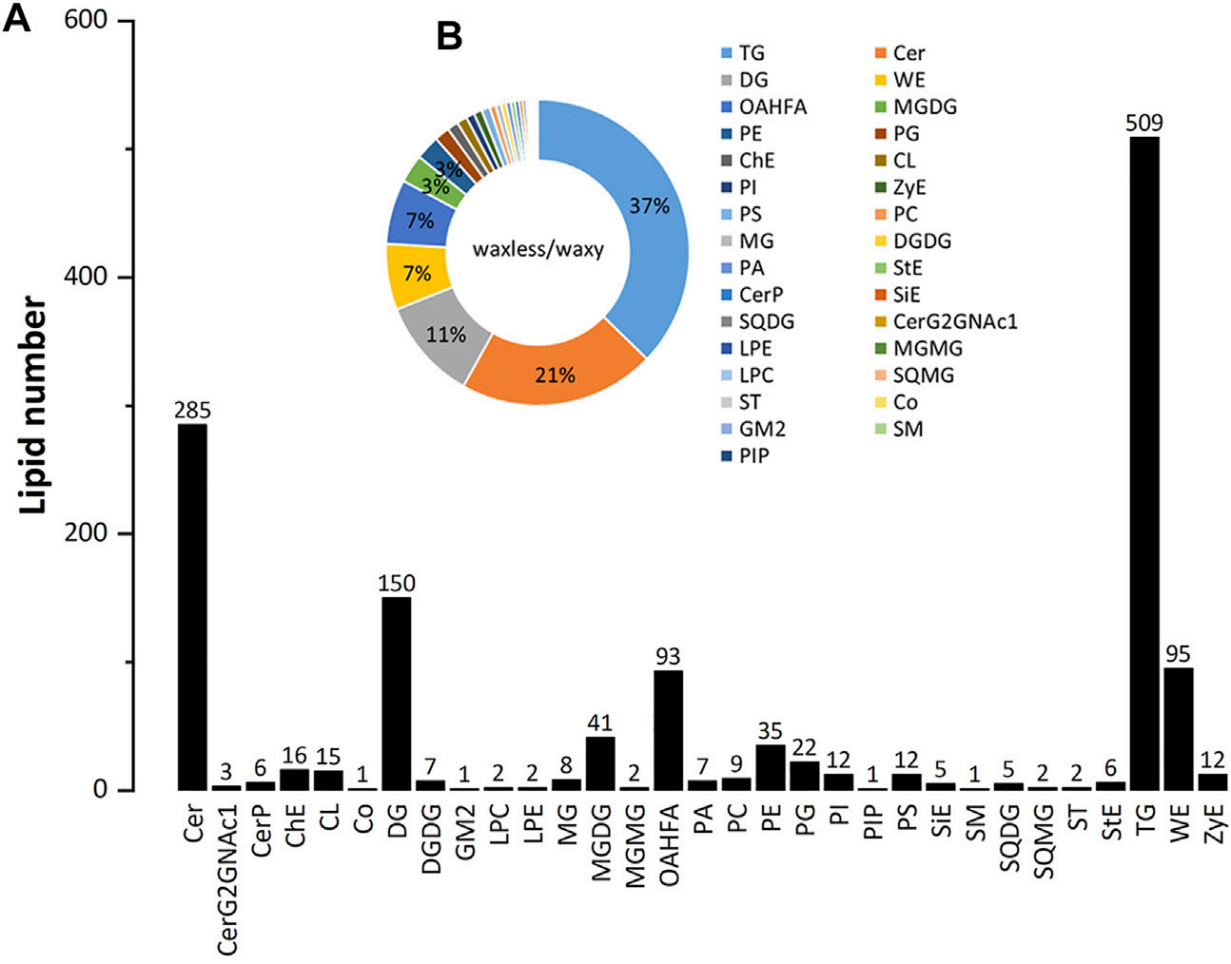
The statistical chart of numbers of lipid subclass in wheat leaves. **(A)** Abscissa is the detected lipid subclasses; ordinate is the number of lipids. **(B)** Different lipid subclasses are represented by different colors, and the proportion is expressed by the size of the color block area. For the corresponding relationship and proportion between each lipid subclass and color, see the legend on the right.

Positive and negative ion patterns of electrospray ionization (ESI) were used in this study. By UPLC analysis, 31 lipid subclasses and 1,367 lipid molecules were identified in the leaf epidermis of the two mutants (Figure 3A). The lipids with a high number of lipid species are triacylglycerol (TG), ceramide (Cer), diacylglycerol (DG), wax ester (WE), (O-acyl)-1-hydroxy fatty acid (OAHFA), monogalactosyl diacylglycerol (MGDG), phosphatidylethanolamine (PE), phosphatidylglycerol (PG), cholesterol (ChE), cardiolipin (CL), phosphatidylinositol (PI), and phosphatidylserine (PS). Among them, TG, Cer, DG, WE, and OAHFA account for 83% of the total number of molecules (Figure 3B). Moreover, it was found that the main lipid components of wheat leaf epidermis were TG, Cer, DG, WE, and OAHFA.

### Analysis of Wax-Related Lipid Subclasses in Leaf Epidermis.

**FIGURE 4:**
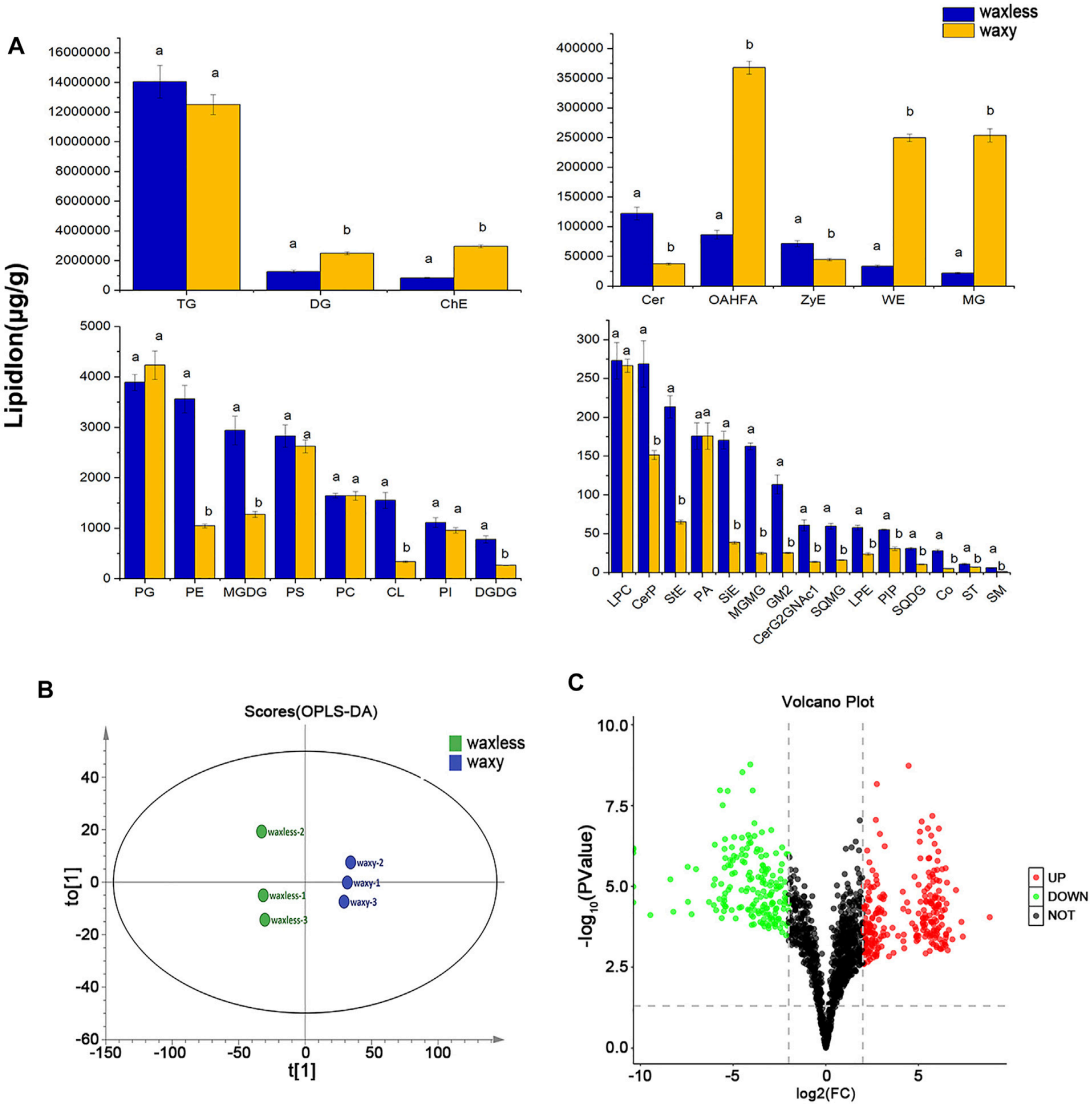
Differential expression of wax-related lipids between *waxless* and *waxy*. **(A)** Content of lipid subclasses in the leaf epidermis of two wheat mutants, abscissa is the lipid subclass, ordinate is the lipid subclass content, blue is the low-wax mutant *waxless*, yellow is the multi-wax mutant *waxy*, and the letters indicate significant differences (*p* < 0.05). **(B)** OPLS-DA score map, “t[1]” represents principal component 1, “to[1]” represents principal component 2, and the ellipse represents 95% confidence interval. Dots of the same color represent each biological weight in the group *waxles*s and *waxy*. **(C)** Volcano plot, abscissa is log_2_FC (*waxless*/*waxy*) and ordinate is −log_10_ (*p* value). Each dot represents a lipid ion, red represents upregulated lipid ions, green represents downregulated lipid ions, and black indicates undifferentiated lipid ions (FC > 4 or < 0.25, VIP>1, *p* < 0.05).

To test the repeatability of samples, we performed OPLS-DA analysis on all samples (Figure 4B). Each group of samples gathered closely and located in the middle of each group, indicating the liposome analysis showed high reproducibility. By comparing the contents of lipid subclasses between low-wax mutants and multi-wax mutants at the heading stage, significant differences in lipids were screened out. As shown in Figure 4A, 11 kinds of lipid substances with significant differences were found between the two mutants (VIP >1, *p* < 0.05). Among them, there are six lipid subclasses with high lipid content in *waxless*, namely, Cer, ZyE, PE, MGDG, CL, and digalactosyl diacylglycerol (DGDG). On the other hand, in *waxy*, five kinds of lipid classes were found to be DG, ChE, OAHFA, WE, and monoacylglycerol (MG) (VIP >1, *p* < 0.05). We infer that the more abundant and different lipids in the *waxy* are the main components of the wax in the epidermis of wheat compared with the *waxless*. That is the main lipid components of wheat leaf epidermis at the heading stage are DG, ChE, OAHFA, WE, and MG.

### Analysis of Lipid Ions Differences in Leaf Epidermis

We analyzed the differences of 1,367 detected lipid ions (FC > 4 or <0.25, VIP >1, *p* < 0.05). Compared with the mutant *waxy*, the mutant *waxless* downregulated 177 lipid ions and upregulated 246 lipid ions. The results were shown in the form of a volcanic map (Figure 4C). 177 lipid ions related to wax metabolism in the epidermis of wheat leaves were screened (FC < 0.25, VIP >1, *p* < 0.05), belonging to eight lipid subclasses, including WE (C19-C50), DG (C27-C53), TG (C29-C73), MG (C31-C35), OAHFA (C31-C52), Cer (C29-C48), ChE (C_46_H_86_O_2_N_1_), and CL (C_65_H_120_O_17_P_2_) (Supplementary Table S2).

The main differential lipid ions in DG are DG(45:2)+H, DG(28:0e)+H and DG (37:4e)+H; ChE(19:0)+NH_4_ in ChE; OAHFA(16:0_31:1)-H, OAHFA(30:1)-H, and OAHFA(31:0)-H in OAHFA; WE(24:0_14:1)+H, WE(28:1_22:0)+H, WE(28:1_18:0)+H, WE(26:0_21:3)+H, and WE(12:0_19:3)+NH_4_ in WE; MG(32:1)+NH_4_, MG(28:1)+H, and MG(32:1)+NH_4_ in MG (Table 1). These may be the main lipid ions that affect the gloss phenotype of wheat leaf epidermis.

**TABLE 1:** Some significant differences in lipid ions between the two mutants.

Class	Lipid ion	Ion formula	Calmz	RT-(min)	VIP	FC(wxless/waxy)	*p* value
TG	TG (16:0e_11:3_16:0)+H	C46 H85 O5	717.639	11.087	1.039	0.0000	1.013E-05
TG	TG(12:1e_16:1_16:1)+H	C47 H87 O5	731.655	11.318	1.038	0.0000	7.176E-07
TG	TG(16:1e_12:2_17:1)+H	C48 H87 O5	743.655	13.602	1.039	0.0030	6.028E-06
TG	TG(16:0e_11:2_16:0)+H	C46 H87 O5	719.655	14.009	1.037	0.0033	6.086E-05
TG	TG(12:0e_16:0_18:4)+H	C49 H89 O5	757.670	16.761	1.037	0.0058	2.997E-05
DG	DG(45:2)+H	C48 H91 O5	747.686	15.278	1.039	0.0000	6.485E-07
DG	DG(28:0e)+H	C31 H63 O4	499.472	5.151	1.039	0.0000	9.089E-07
DG	DG(37:4e)+H	C40 H73 O4	617.550	15.722	1.038	0.0000	3.125E-05
DG	DG(44:3e)+H	C47 H89 O4	717.676	16.783	1.035	0.0014	7.660E-05
DG	DG(34:1e)+NH_4_	C37 H76 O4 N1	598.577	6.326	1.037	0.0057	2.469E-06
Cer	Cer(d16:1_15:0)+H	C31 H62 O3 N1	496.472	6.224	1.037	0.0152	3.802E-05
Cer	Cer(m17:1_12:0)+H	C29 H58 O2 N1	452.446	8.725	1.038	0.0187	2.452E-05
Cer	Cer(d19:1_12:0)+H	C31 H62 O3 N1	496.472	8.162	1.036	0.0204	9.540E-05
Cer	Cer(d15:0_16:1)+H	C31 H62 O3 N1	496.472	5.816	1.037	0.0237	4.217E-05
Cer	Cer(m46:0)+H	C46 H94 O2 N1	692.728	15.058	1.039	0.0302	4.069E-06
ChE	ChE(19:0)+NH_4_	C46 H86 O2 N1	684.665	15.942	1.036	0.0296	5.379E-05
CL	CL(56:2)-2H	C65 H120 O17 P2	617.401	7.945	1.037	0.2104	3.561E-05
OAHFA	OAHFA(16:0_31:1)-H	C47 H89 O4	717.677	16.781	1.036	0.0158	5.938E-06
OAHFA	OAHFA(30:1)-H	C30 H55 O4	479.411	10.509	1.037	0.0166	5.445E-05
OAHFA	OAHFA(31:0)-H	C31 H59 O4	495.442	6.295	1.039	0.0200	8.356E-06
OAHFA	OAHFA(15:0_16:0)-H	C31 H59 O4	495.442	8.630	1.038	0.0212	3.053E-08
OAHFA	OAHFA(31:1)-H	C31 H57 O4	493.426	21.881	1.037	0.0281	1.509E-06
MG	MG(32:1)+NH_4_	C35 H72 O4 N1	570.546	6.394	1.038	0.0216	3.395E-05
MG	MG(28:1)+H	C31 H61 O4	497.456	6.322	1.038	0.0228	2.612E-06
MG	MG(32:1)+NH_4_	C35 H72 O4 N1	570.546	8.707	1.035	0.0345	9.770E-05
WE	WE(24:0_14:1)+H	H75 C38 O2	563.576	15.814	1.039	0.0231	3.439E-07
WE	WE(28:1_22:0)+H	H99 C50 O2	731.764	21.557	1.037	0.0260	2.186E-06
WE	WE(28:1_18:0)+H	H91 C46 O2	675.701	19.485	1.039	0.0299	6.087E-07
WE	WE(26:0_21:3)+H	H89 C47 O2	685.686	16.346	1.039	0.0322	1.128E-06
WE	WE(12:0_19:3)+NH_4_	H60 C31 O2 N1	478.462	6.817	1.037	0.0425	4.349E-05

Note: triacylglycerol (TG), diacylglycerol (DG), ceramide (Cer), cardiolipin (CL), (O-acyl)-1-hydroxy fatty acid (OAHFA), wax ester (WE), and monoacylglycerol (MG).

### Analysis of Carbon Chain Length of Differential Lipids

**FIGURE 5:**
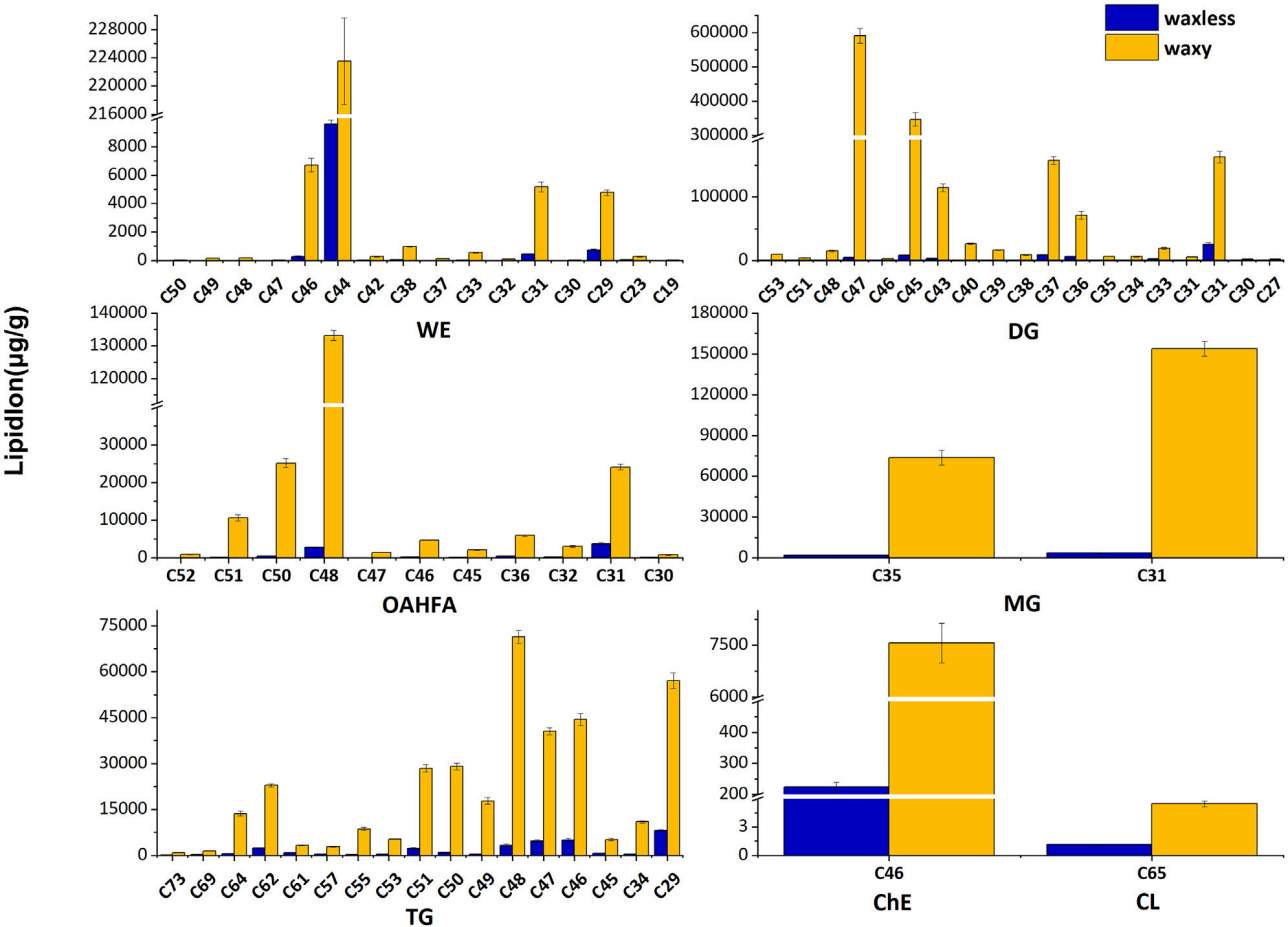
Carbon chain length distribution. Abscissa denotes the lipid molecules with different carbon chain lengths, ordinate indicates lipid ion content, blue is the low-wax mutant *waxless*, and yellow is the multi-wax mutant *waxy*.

In addition to the content of lipids, the carbon chain length of lipids is also an important factor that cannot be ignored. We added the lipid ions with the same carbon chain length and counted the lipid ions with diverse carbon chain lengths under different lipid subclasses to further analyze the main discrepant lipids in wheat epidermis wax (Figure 5). Compared with *waxy* mutants, the lipid ions significantly decreased in *waxless* mutants were WE (C29), WE (C31), WE (C44), WE (C46), DG (C47), DG (C45), DG (C43), DG (C37), DG (C36), DG (C31), OAHFA (C51), OAHFA (C50), OAHFA (C48), OAHFA (C31), MG (C35), MG (C31), and TG (C64).

These lipid ions affect the synthesis, transcription, and transport of wax through the change of carbon chain length, leading to the smooth green phenotype of wheat waxy deletion mutants. We found that WE in wheat leaf epidermis is mainly concentrated in C44-dominated wax ester molecules and also widely distributed in C46, C31, and C29 wax ester molecules but less in other chain lengths. DG in the wax mixture of the wheat epidermis is mainly concentrated in C47, C45, C37, and C31 molecules. In OAHFA, C48 plays a leading role, accompanied by a large number of C50-C51 and C31 molecules. C35 and C31 play significant roles in MG. On the other hand, in TG, the length of the carbon chain varies from C29 to C64, in which C48 plays a major role.

### Gene Differential Expression Analysis

**FIGURE 6:**
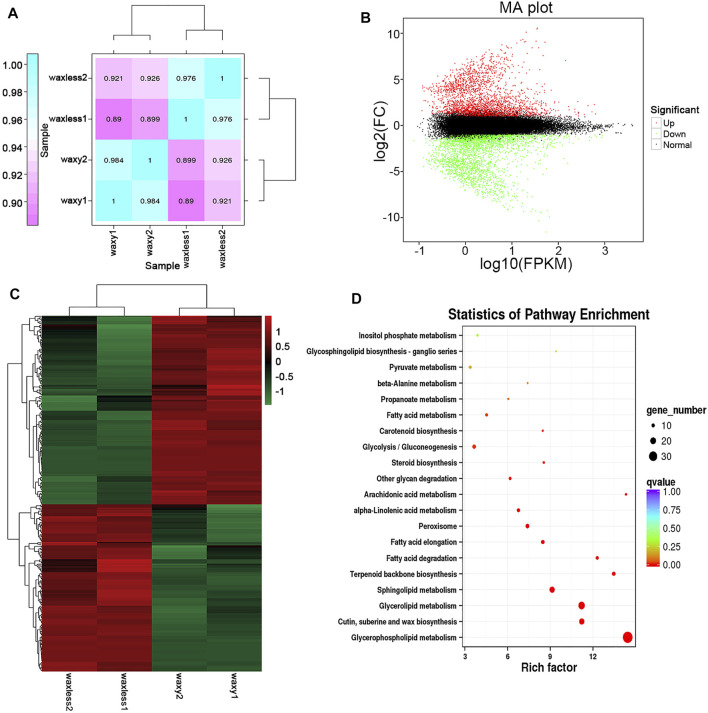
Transcriptome data analysis. **(A)** The expression quantity correlation heatmap of the sample. The numbers in each cell represent the correlation coefficients. **(B)** MA plot of DEGs, each dot in the MA map of differentially expressed genes represents a gene. Abscissa is value A: log_10_ (FPKM), ordinate is value M: log_2_FC(*waxless*/*waxy*), green dots represent the downregulated DEGs, red dots represent the upregulated DEGs, and black dots represent the non-differentially expressed genes. **(C)** The heatmap presentation of fold changes of 343 DEGs obtained from RNA-seq analysis. **(D)** Enrichment and scatter map of KEGG pathway of DEGs. Each circle in the figure represents a KEGG pathway, ordinate represents the name of the pathway, and abscissa is the enrichment factor. The greater the enrichment factor, the more significant the enrichment level of DEGs in this pathway. The color of the circle represents the qvalue. The size of the circle indicates the number of genes enriched in the pathway, and the larger the circle, the more the genes.

Through the analysis of leaf transcriptional groups of low-wax mutants and multi-wax mutants at the heading stage, correlation coefficients among different biological repetitive samples ranged from 0.890 to 0.926 (Figure 6A). Compared with the mutant *waxy*, 6,840 DEGs were detected in the mutant *waxless*, of which 3,181 genes were upregulated and 3,659 genes were downregulated (Fold Change ≥2, FDR <0.01) (Figure 6B). To understand the biological significance between low-wax mutant and multi-wax mutant DEGs, these genes were enriched by Gene Ontology (GO) analysis. We found that the biological processes were mainly enriched in the metabolic process (28.89%), cellular process (24.73%), single-organism process (14.96%), biological regulation (8.35%), and response to stimulus (6.46%). These genes are mainly distributed in the membrane, membrane part, cell, component cell part, and organelle. The molecular functions mainly include binding and catalytic activity (Supplementary Figure S1).

Furthermore, we annotated the lipid function of 3,659 DEGs, screened 343 DEGs, and enriched them using the Kyoto Encyclopedia of Genes and Genomes (KEGG) enrichment analysis (Figures 6C,D). It was found that these genes are mainly concentrated in glycerophospholipid metabolism, cutin, suberine and wax biosynthesis, glycerolipid metabolism, sphingolipid metabolism, terpenoid backbone biosynthesis, fatty acid degradation, and fatty acid elongation pathway, which are consistent with the main lipid components that control the glossy phenotype of wheat leaves in this study.

### Joint Analysis of Waxy and Differentially Expressed Genes

**FIGURE 7:**
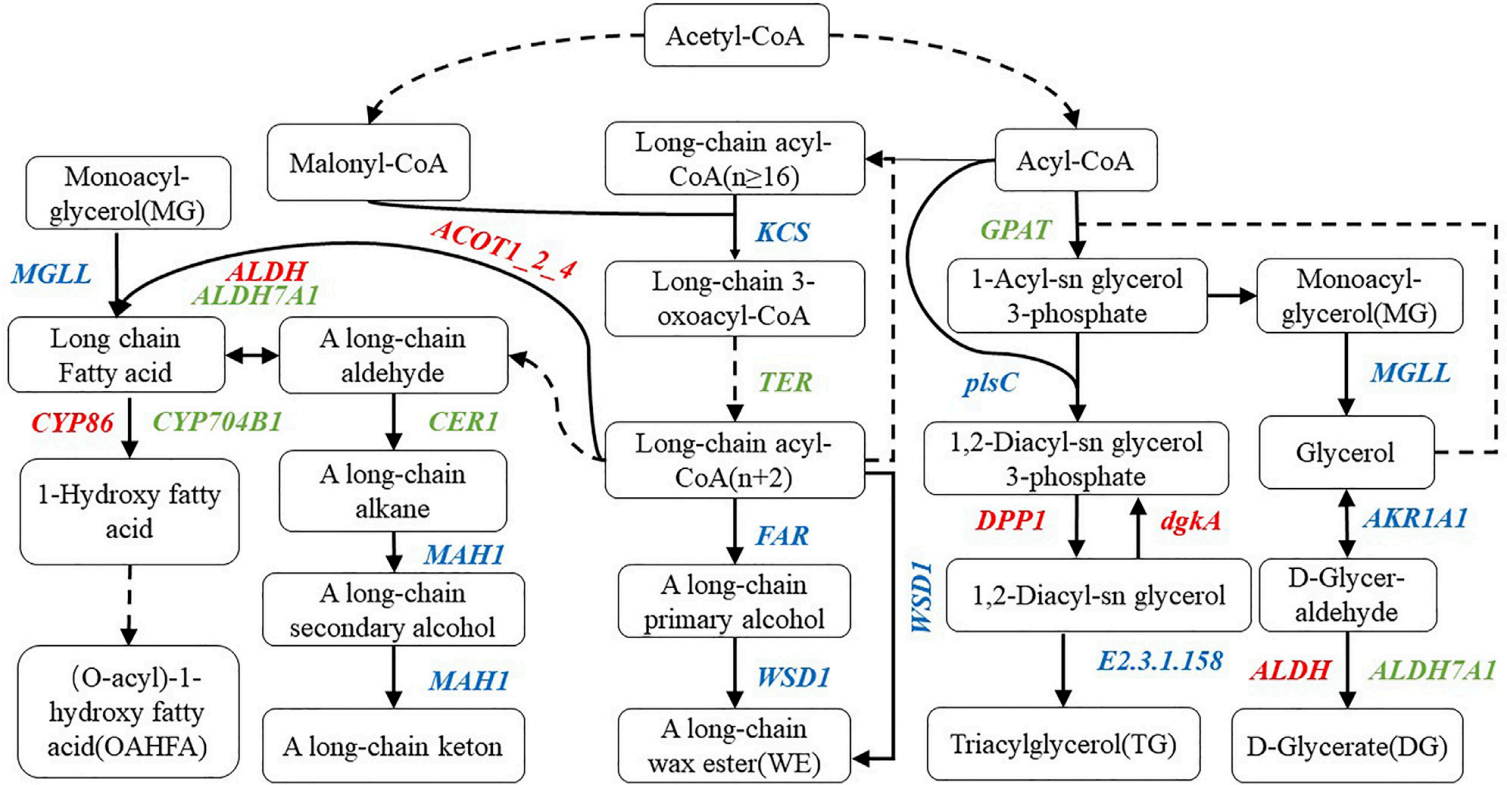
Metabolic pathway map of wax-related lipid synthesis in wheat epidermis. There are metabolic compounds in the box, red-marked enzymes are related to upregulated genes, green-marked enzymes are related to downregulated genes, and blue-marked enzymes are related to both upregulated and downregulated genes.

Combined with the determined lipid components, we marked the annotated genes in the corresponding pathway, constructed the metabolic model of the main lipid components in wheat epidermis according to the KEGG metabolic pathway (Figure 7), and screened 46 related genes (Table 2). Moreover, compared with the multi-wax mutant *waxy*, the genes annotated to the synthesis of wax ester (WE) in the low-wax mutant *waxless* were 3-ketoacyl-CoA synthase genes (*KCS*), very-long-chain enoyl-CoA reductase genes (*TER*), alcohol-forming fatty acyl-CoA reductase genes (*FAR*), wax-ester synthase genes (*WSD1*), aldehyde decarbonylase genes (*CER1*), and midchain alkane hydroxylase genes (*MAH1*). The downregulation of *TER* inhibits the conversion of long-chain 3-oxoacyl-CoA to long-chain acyl-CoA (n + 2), the downregulation of *CER1* inhibits the transformation of upstream A long-chain aldehyde to A long-chain alkane, and the expression genes involved in *KCS*, *FAR*, *WSD1*, and *MAH1* were both upregulated and downregulated.

**TABLE 2:** The differentially expressed genes related to epidermal wax synthesis pathway in wheat.

Gene ID	log2FC (*waxless*/*waxy*)	Developing	KEGG orthology	Gene symbol	Definition
TraesCS1D03G0373900	−3.361317375	Down	K15404	*CER1*	Aldehyde decarbonylase
TraesCS1D03G0374000	−2.240488466	Down	K15404	*CER1*	Aldehyde decarbonylase
TraesCS3B03G0017600	−2.077102639	Down	K15406	*WSD1*	Wax-ester synthase
TraesCS3B03G0037000	−3.159781364	Down	K15406	*WSD1*	Wax-ester synthase
TraesCS5D03G0740400	−1.28631545	Down	K15406	*WSD1*	Wax-ester synthase
TraesCS5A03G0782000	5.129181799	Up	K15406	*WSD1*	Wax-ester synthase
TraesCS5B03G0814100	2.492795173	Up	K15406	*WSD1*	Wax-ester synthase
TraesCS3B03G0228000	−1.137168478	Down	K20495	*CYP704B1*	Long-chain fatty acid omega-monooxygenase
TraesCS3B03G0938500	−1.18751546	Down	K20495	*CYP704B1*	Long-chain fatty acid omega-monooxygenase
TraesCS3D03G0167200	−2.160568355	Down	K20495	*CYP704B1*	Long-chain fatty acid omega-monooxygenase
TraesCSU03G0103800	1.359012742	Up	K15405	*MAH1*	Midchain alkane hydroxylase
TraesCS4A03G1162200	−7.768462261	Down	K15405	*MAH1*	Midchain alkane hydroxylase
TraesCS1D03G0041600	5.136041959	Up	K13356	*FAR*	Alcohol-forming fatty acyl-CoA reductase
TraesCS4B03G0019500	−1.972216449	Down	K13356	*FAR*	Alcohol-forming fatty acyl-CoA reductase
TraesCS7B03G1338900	−1.447125679	Down	K13356	*FAR*	Alcohol-forming fatty acyl-CoA reductase
TraesCS6B03G0695700	2.649123598	Up	K15402	*CYP86*	Fatty acid omega-hydroxylase
TraesCS2B03G1160500	−1.725507817	Down	K00655	*plsC*	1-acyl-sn-glycerol-3-phosphate acyltransferase
TraesCS4A03G1087000	4.199580543	Up	K00655	*plsC*	1-acyl-sn-glycerol-3-phosphate acyltransferase
TraesCS6A03G0010400	2.48094237	Up	K00655	*plsC*	1-acyl-sn-glycerol-3-phosphate acyltransferase
TraesCS4B03G0110200	1.172254409	Up	K01054	*MGLL*	Acylglycerol lipase
TraesCS4B03G0111500LC	−6.217652748	Down	K01054	*MGLL*	Acylglycerol lipase
TraesCS6B03G0004500	5.978405721	Up	K00128	*ALDH*	Aldehyde dehydrogenase (NAD+)
TraesCS5B03G0557800	−1.393721239	Down	K14085	*ALDH7A1*	Aldehyde dehydrogenase family 7 member A1
TraesCS5D03G0511400	−1.556106189	Down	K14085	*ALDH7A1*	Aldehyde dehydrogenase family 7 member A1
TraesCS6A03G0083200	−1.967010361	Down	K13508	*GPAT*	Glycerol-3-phosphate acyltransferase
TraesCS6B03G0120600	−1.672710452	Down	K13508	*GPAT*	Glycerol-3-phosphate acyltransferase
TraesCS4A03G0991200	1.398499657	Up	K00679	*E2.3.1.158*	Phospholipid:diacylglycerol acyltransferase
TraesCS6A03G0788900	1.856281232	Up	K00679	*E2.3.1.158*	Phospholipid:diacylglycerol acyltransferase
TraesCS7A03G0874000	−1.849596941	Down	K00679	*E2.3.1.158*	Phospholipid:diacylglycerol acyltransferase
TraesCS1A03G0712300	3.228966834	Up	K00002	*AKR1A1*	Alcohol dehydrogenase (NADP+)
TraesCS3B03G0102700	1.348166602	Up	K00002	*AKR1A1*	Alcohol dehydrogenase (NADP+)
TraesCS3B03G0987300	−1.189917126	Down	K00002	*AKR1A1*	Alcohol dehydrogenase (NADP+)
TraesCS7D03G0536500	−1.885493349	Down	K00002	*AKR1A1*	Alcohol dehydrogenase (NADP+)
TraesCS3D03G0571200	1.44849726	Up	K18693	*DPP1*	Diacylglycerol diphosphate phosphatase
TraesCS2A03G1233700	1.054654678	Up	K00901	*dgkA*	Diacylglycerol kinase (ATP)
TraesCS1A03G1066900	−6.470126296	Down	K15397	*KCS*	3-ketoacyl-CoA synthase
TraesCS4B03G0018800LC	−3.017642109	Down	K15397	*KCS*	3-ketoacyl-CoA synthase
TraesCS6A03G0053200	−2.314714201	Down	K15397	*KCS*	3-ketoacyl-CoA synthase
TraesCS6D03G0655900	−2.157514317	Down	K15397	*KCS*	3-ketoacyl-CoA synthase
TraesCS1B03G1176600	1.132972231	Up	K15397	*KCS*	3-ketoacyl-CoA synthase
TraesCS6A03G0162400	1.030585291	Up	K15397	*KCS*	3-ketoacyl-CoA synthase
TraesCS7A03G0089600LC	3.358707584	Up	K15397	*KCS*	3-ketoacyl-CoA synthase
TraesCS7A03G0089800LC	3.35205506	Up	K15397	*KCS*	3-ketoacyl-CoA synthase
TraesCS7D03G0081000	1.589752288	Up	K15397	*KCS*	3-ketoacyl-CoA synthase
TraesCS6B03G1132900LC	−4.230220979	Down	K10258	*TER*	Very-long-chain enoyl-CoA reductase
TraesCS2B03G1089600	1.12980861	Up	K01068	*ACOT1_2_4*	Acyl-coenzyme A thioesterase 1/2/4

In the process of OAHFA synthesis, the upregulated expression of acyl-coenzyme A thioesterase 1/2/4 genes (*ACOT1_2_4*) promotes the synthesis of long-chain fatty acid, while the expression level of acylglycerol lipase genes (*MGLL*) was both upregulated and downregulated during the conversion of monoacyl-glycerol to long-chain fatty acid. Downregulation of aldehyde dehydrogenase family 7 member A1 genes (*ALDH7A1*) expression inhibited the transition between A long-chain aldehyde and long-chain fatty acid, while aldehyde dehydrogenase genes (*ALDH*) upregulated the transition between them. During the transformation of long-chain fatty acid into omega-hydroxy fatty acid, the expression of long-chain fatty acid omega-monooxygenase genes (*CYP704B1*) was downregulated, while that of fatty acid omega-hydroxylase genes (*CYP86*) was upregulated in the mutant *waxless*. Glycerol-3-phosphate acyltransferase genes (*GPAT*) and *ALDH7A1* were downregulated in the process of glyceride metabolism of DG, MG, and TG. The downregulated expression of *GPAT* inhibits the synthesis of 1-acyl-sn glycerol 3-phosphate in *waxless*, which in turn reduces the synthesis of MG. The downregulated expression of *ALDH7A1* in the low-wax mutant decreases the synthesis of DG. The upregulated genes are *ALDH*, diacylglycerol diphosphate phosphatase genes (*DPP1*), and diacylglycerol kinase genes (*dgkA*), while the expressions of 1-acyl-sn-glycerol-3-phosphate acyltransferase genes *(plsC*), phospholipid: diacylglycerol acyltransferase genes (*E2.3.1.158*), *MGLL*, and alcohol dehydrogenase genes (*AKR1AI*) were both upregulated and downregulated. These genes work together to reduce the content of TG in the low-wax mutation *waxless*. In order to verify the reliability of our data for screening wax-related genes, we randomly selected 13 differentially expressed genes for real-time quantitative PCR detection, and the results of qRT-PCR of 13 genes were consistent with those of transcriptome data (Supplementary Figure S2), indicating that the screened wax-related genes are highly reliable, which is helpful for further functional verification and cloning of wax-related genes.

## Discussion

### New Waxy Components of Wheat Epidermis Were Found by Untargeted Lipidomic Analysis

Epidermal wax is a complex lipid mixture composed of VLCFAs and their derivatives, which plays a special role in plant resistance to drought, diseases, and insect pests (Bernard and Joubes, 2013). At present, the GC-MS method is mainly used to determine the wax of epidermis (Rolim et al., 2015) and primary alcohols, secondary alcohols, aldehydes, alkanes, ketones, esters, triterpenes, sterols, and flavonoids (Zhang et al., 2005; Tafolla-Arellano et al., 2018). Based on the limitations of the determination method, there may still be many epidermal wax-related lipid components that have not been detected, which limits the study of the epidermal wax biosynthesis pathway. Lipidomics as a research model based on high-throughput analysis can systematically analyze the changes in lipid composition and expression in organisms. Perez-Navarro et al. (2019) used LC-MS technology to determine grape lipids and found new information on the composition of free fatty acids such as glycerol, glycerol phospholipids, and triterpenes in grape skins and seeds. Broughton et al. (2018) used electrospray ionization tandem mass spectrometry (ESI-MS/MS) to analyze the acyl parts of wax esters (WE) and sterol esters (SE) in common and mutant sunflower oils with different fatty acid profiles and discovered the methylsterol components in sunflower oil sterol esters (SE) for the first time.

Bianchi et al. (1980) studied the epidermal wax of common wheat in Chinese spring and found that its main components are n-alkanes, esters, aldehydes, free alcohols, β-diketones and hydroxy-β-diketones. Racovita et al. (2016) detected new waxy substances such as 2-alkyl alcohol, benzyl alcohol, phenylethyl alcohol, and hydroxyphenylethanol in wheat flag leaves and peduncles. Lavergne et al. (2018) analyzed the epidermis of wheat leaves and stems and found that the waxy components of the epidermis were alkanes (C20-C42), fatty acids (C7-C34), ketones (C9-C35), and primary alcohols (C22-C33). In this study, UHPLC-MS/MS analysis technique was used for the first time to detect the leaf epidermis wax of low-wax mutant *waxless* and multi-wax mutant *waxy* of Jimai 22. We found that the main lipid components of leaf epidermis wax of Jimai 22 were WE (C19-C50), DG (C27-C53), MG (C31-C35), OAHFA (C31-C52), TG (C29-C73), Cer (C29-C48), ChE (C_46_H_86_O_2_N_1_), and CL (C_65_H_120_O_17_P_2_). Among them, DG, MG and OAHFA were detected in wheat leaf wax for the first time, which improved the composition of wheat wax components. It is clear that the liposome analysis of wheat epidermis wax by LC-MS can more systematically study the changes and functions of related lipid subclasses and lipid molecules in the process of epidermal wax metabolism, which is helpful to improve the pathway and mechanism of epidermal wax synthesis.

### New Components Such as Diacylglycerol, Monoacylglycerol, and (O-Acyl)-1-hydroxy Fatty Acid Are Closely Related to Stress Resistance

In this research, we found that the wax content was closely related to the water loss rate of leaves, which may be related to drought resistance (Zhang et al., 2013). Besides, DG, MG, and OAHFA were newly found in wheat epidermis wax. DG, as one of the main components of glycerol, plays a critical role in maintaining the stability of cell membrane (especially plasma membrane and chloroplast membrane) under high temperature and drought stress (Narayanan et al., 2016). It can maintain the fluidity of cell membrane by downregulating saturated DGs (4 and 10 double bonds) and u-regulating low unsaturated DGs (0–4 double bonds) (Navarro-Reig et al., 2019). MG, as a waxy substance, can form stable hydrated dispersions in water. Under salt stress, the ratio of MG unsaturated fatty acids to saturated fatty acids increased significantly in salt-tolerant varieties (Gogna et al., 2020). The MG in wheat epidermis wax found in this study is also mainly MG (32:1) + NH_4_ and MG (28:1) + H with low saturation. OAHFA acts as a surfactant in human tear film lipids and plays a key role in stabilizing tear film (Schuett and Millar, 2013; Marshall et al., 2016). Interestingly, we found OAHFA in wheat leaf epidermis wax for the first time and C48 molecules play a leading role. Its effects on wheat stress resistance and related mechanism need to be further studied.

In addition, as an important oil substance, WE plays a significant role in plant resistance to drought because of its hydrolytic resistance (Ivarson et al., 2017; Wang et al., 2021). The WE wax found by predecessors in sunflower is mainly concentrated in C32–C48 molecules (Broughton et al., 2018). In our study, we found that the WE wax in the epidermis of wheat leaves is mainly distributed in the molecular range of C29–C46 chain length, and the wax ester molecule dominated by C44 plays a role, which is similar to the results of previous studies.

### Molecular Regulation Mechanism of Epidermal Wax Metabolism in Wheat

Previous studies have shown that the *W1-W5* homologue of *Arabidopsis CER* protein in wheat increases the wax content of the epidermis by producing hydroxy-β-diketone and inducing the biosynthesis of β-diketone (Huang et al., 2017; Li et al., 2020). In this study, two *CER1* genes, *TraesCS1D03G0373900* and *TraesCS1D03G0374000*, were also detected in the low-wax mutants, which decreased the synthesis of A long-chain alkane by downregulating the expression of *CER1*. *TaFAR1*, *TaFAR2*, *TaFAR3*, *TaFAR4*, *TaFAR5,* and *AtCER4* are homologous genes of *CER4* in *Arabidopsis*. As a kind of alcohol-forming fatty acyl-CoA reductase, they induce the production of A long-chain primary alcohol and increase the wax content of wheat leaves (Wang W. et al., 2015; Wang et al., 2015b; Wang et al., 2015c; Wang et al., 2017). Besides, two *FAR* genes regulating primary alcohol synthesis, *TraesCS4B03G0019500,* and *TraesCS7B03G1338900*, were also found in the mutant *waxless*, which downregulated primary alcohol synthesis and promoted the smooth green appearance of the waxy wheat epidermis. In the research of low-wax mutants of wheat, Li et al. (2021) found that the expression levels of *ACC1*, *LACS*, *KCS,* and *KCR* were downregulated, which reduced the synthesis of VLCFAs. In our research, four *KCS* genes were also found to downregulate long-chain 3-oxoacyl-CoA synthesis in *waxless*. In addition, we found *TER* gene *TraesCS6B03G1132900LC* in wheat mutant *waxless* leaves for the first time, which inhibited the synthesis of long-chain acyl-CoA (n + 2) by downregulating its expression. This provides important reference information for enriching the molecular regulation mechanism of wheat epidermis wax synthesis.

Moreover, our study also found that the downregulation of two *ALDH7A1* genes *TraesCS5B03G0557800* and *TraesCS5D03G0511400* inhibited the synthesis of long-chain fatty acid and DG, the downregulation of three *CYP704B1* genes *TraesCS3B03G0228000*, *TraesCS3B03G0938500,* and *TraesCS3D03G0167200* inhibited the synthesis of omega-hydroxy fatty acid, and the downregulation of *GPAT* genes *TraesCS6A03G0083200* and *TraesCS6B03G0120600* downregulated the expression of 1-acyl-sn glycerol 3-phosphate in DG, MG, and TG during glyceride metabolism, which inhibited the synthesis of 1-acyl-sn glycerol 3-phosphate in wax-free mutants. The downregulated expression of *plsC* gene *TraesCS2B03G1160500* reduces the synthesis of 1,2-diacyl-sn glycerol 3-phosphate. Downregulation of phospholipid: diacylglycerol acyltransferase genes *TraesCS7A03G0874000* expression inhibits the transformation from 1,2-diacyl-sn glycerol to TG. These genes are found for the first time to regulate waxy synthesis in the wheat epidermis. In-depth genomic comparison and functional identification of these genes are helpful in cloning wheat waxy functional genes and analyzing the regulatory mechanism of waxy metabolism. Our next research will focus on the functional verification of key genes to better explain the molecular regulation mechanism of wax in the leaf epidermis of wheat.

## Conclusion

In this study, untargeted liposome detection and transcriptome analysis were carried out on the leaf epidermis wax of Jimai 22 low-wax mutant and multi-wax mutant. A total of 31 lipid subclasses and 1,367 lipid molecules were identified. The main lipid components of wheat leaf wax were identified as WE (C19-C50), DG (C27-C53), TG (C29-C73), MG (C31-C35), and OAHFA (C31-C52). DG, MG, and OAHFA were found in the epidermis wax of wheat leaf for the first time. Compared with the mutant *waxy*, a total of 6,840 DEGs were detected in the mutant *waxless*, of which 3,181 DEGs were upregulated and 3,659 DEGs were downregulated. According to KEGG metabolic pathway, the metabolic pattern of the main waxy components in the wheat epidermis was constructed and 46 related genes were screened, including *KSC*, *TER*, *FAR*, *WSD1*, *CER1*, *MAH1*, *ALDH7A1*, *CYP704B1*, *ACOT1_2_4*, *ALDH*, *CYP86*, MGLL, *GPAT*, *DPP1*, *dgkA*, *plsC,* and *E2.3.1.158* related genes. This provides valuable reference information for further study of wheat epidermis wax inheritance and molecular regulation.

## Data Availability

The datasets presented in this study can be found in online repositories. The names of the repository/repositories and accession number(s) can be found in the article/Supplementary Material. Mass spectrometry data has been uploaded to EBI Metabolights, accession number MTBLS3710. Sequencing data has been uploaded to NCBI SRA BioProject, accession number PRJNA777112.

## References

[B1] Aharoni A. Dixit S. Jetter R. Thoenes E. van Arkel G. Pereira A. (2004). The SHINE Clade of AP2 Domain Transcription Factors Activates Wax Biosynthesis, Alters Cuticle Properties, and Confers Drought Tolerance when Overexpressed in Arabidopsis[W]. Plant Cell 16, 2463–2480. 10.1105/tpc.104.022897 15319479 PMC520946

[B2] Bernard A. Joubès J. (2013). *Arabidopsis* Cuticular Waxes: Advances in Synthesis, export and Regulation. Prog. Lipid Res. 52, 110–129. 10.1016/j.plipres.2012.10.002 23103356

[B3] Bianchi G. Lupotto E. Borghi B. Corbellini M. (1980). Cuticular Wax of Wheat. Planta 148, 328–331. 10.1007/bf00388119 24310134

[B4] Bourdenx B. Bernard A. Domergue F. Pascal S. Léger A. Roby D. (2011). Overexpression of Arabidopsis ECERIFERUM1 Promotes Wax Very-Long-Chain Alkane Biosynthesis and Influences Plant Response to Biotic and Abiotic Stresses. Plant Physiol. 156, 29–45. 10.1104/pp.111.172320 21386033 PMC3091054

[B5] Broughton R. Ruíz-Lopez N. Hassall K. L. Martínez-Force E. Garcés R. Salas J. J. (2018). New Insights in the Composition of Wax and Sterol Esters in Common and Mutant sunflower Oils Revealed by ESI-MS/MS. Food Chem. 269, 70–79. 10.1016/j.foodchem.2018.06.135 30100486

[B6] Chai G. Li C. Xu F. Li Y. Shi X. Wang Y. (2018). Three Endoplasmic Reticulum-Associated Fatty Acyl-Coenzyme A Reductases Were Involved in the Production of Primary Alcohols in Hexaploid Wheat (*Triticum aestivum* L.). BMC Plant Biol. 18, 41. 10.1186/s12870-018-1256-y 29506473 PMC5836450

[B7] Dong X. Ji J. Yang L. Fang Z. Zhuang M. Zhang Y. (2019). Fine-mapping and Transcriptome Analysis of *BoGL-3*, a Wax-Less Gene in Cabbage (*Brassica oleracea* L. Var. Capitata). Mol. Genet. Genomics 294, 1231–1239. 10.1007/s00438-019-01577-5 31098741

[B8] Franke R. Briesen I. Wojciechowski T. Faust A. Yephremov A. Nawrath C. (2005). Apoplastic Polyesters in *Arabidopsis* Surface Tissues - A Typical Suberin and a Particular Cutin. Phytochemistry 66, 2643–2658. 10.1016/j.phytochem.2005.09.027 16289150

[B9] Gogna M. Choudhary A. Mishra G. Kapoor R. Bhatla S. C. (2020). Changes in Lipid Composition in Response to Salt Stress and its Possible Interaction with Intracellular Na+-K+ Ratio in sunflower (Helianthus Annuus L.). Environ. Exp. Bot. 178, 104147. 10.1016/j.envexpbot.2020.104147

[B10] Huang D. Feurtado J. A. Smith M. A. Flatman L. K. Koh C. Cutler A. J. (2017). Long Noncoding miRNA Gene Represses Wheat β-diketone Waxes. Proc. Natl. Acad. Sci. USA 114, E3149–E3158. 10.1073/pnas.1617483114 28351975 PMC5393243

[B11] Ivarson E. Iven T. Sturtevant D. Ahlman A. Cai Y. Chapman K. (2017). Production of Wax Esters in the Wild Oil Species *Lepidium Campestre* . Ind. Crops Prod. 108, 535–542. 10.1016/j.indcrop.2017.07.002

[B12] Kannangara R. Branigan C. Liu Y. Penfield T. Rao V. Mouille G. (2007). The Transcription Factor *WIN1/SHN1* Regulates Cutin Biosynthesis in *Arabidopsis thaliana* . Plant cell 19, 1278–1294. 10.1105/tpc.106.047076 17449808 PMC1913754

[B13] Lavergne F. Broeckling C. Cockrell D. Haley S. Peairs F. Jahn C. (2018). GC-MS Metabolomics to Evaluate the Composition of Plant Cuticular Waxes for Four *Triticum aestivum* Cultivars. Ijms 19 (2), 249. 10.3390/ijms19020249 PMC5855543 29360745

[B14] Li L. Qi Z. Chai L. Chen Z. Wang T. Zhang M. (2020). The Semidominant Mutation *W5* Impairs Epicuticular Wax Deposition in Common Wheat (*Triticum aestivum* L.). Theor. Appl. Genet. 133, 1213–1225. 10.1007/s00122-020-03543-x 31965231

[B15] Li L. Zhang Z. Song W. Su Z. Zhang Y. You M. (2021). The Essential Role of *W5* in Wax Metabolism in Wheat (*Triticum aestivum* L.). J. Plant Biol. 10.1007/s12374-021-09325-2

[B16] Marshall D. L. Saville J. T. Maccarone A. T. Ailuri R. Kelso M. J. Mitchell T. W. (2016). Determination of Ester Position in Isomeric (O -Acyl)-Hydroxy Fatty Acids by Ion Trap Mass Spectrometry. Rapid Commun. Mass. Spectrom. 30, 2351–2359. 10.1002/rcm.7715 27520617

[B17] Narayanan S. Tamura P. J. Roth M. R. Prasad P. V. V. Welti R. (2016). Wheat Leaf Lipids during Heat Stress: I. High Day and Night Temperatures Result in Major Lipid Alterations. Plant Cel Environ. 39, 787–803. 10.1111/pce.12649 PMC5102054 26436679

[B18] Navarro-Reig M. Tauler R. Iriondo-Frias G. Jaumot J. (2019). Untargeted Lipidomic Evaluation of Hydric and Heat Stresses on rice Growth. J. Chromatogr. B 1104, 148–156. 10.1016/j.jchromb.2018.11.018 30471516

[B19] Pérez-Navarro J. Da Ros A. Masuero D. Izquierdo-Canas P. M. Hermosín-Gutiérrez I. Gomez-Alonso S. (2019). LC-MS/MS Analysis of Free Fatty Acid Composition and Other Lipids in Skins and Seeds of *Vitis vinifera* Grape Cultivars. Food Res. Int. 125, 108556. 10.1016/j.foodres.2019.108556 31554044

[B20] Pizarro C. Arenzana-Rámila I. Pérez-del-Notario N. Pérez-Matute P. González-Sáiz J.-M. (2013). Plasma Lipidomic Profiling Method Based on Ultrasound Extraction and Liquid Chromatography Mass Spectrometry. Anal. Chem. 85, 12085–12092. 10.1021/ac403181c 24266777

[B21] Racovita R. C. Hen-Avivi S. Fernandez-Moreno J.-P. Granell A. Aharoni A. Jetter R. (2016). Composition of Cuticular Waxes Coating Flag Leaf Blades and Peduncles of *Triticum aestivum* Cv. Bethlehem. Phytochemistry 130, 182–192. 10.1016/j.phytochem.2016.05.003 27264640

[B22] Reina-Pinto J. J. Yephremov A. (2009). Surface Lipids and Plant Defenses. Plant Physiol. Biochem. 47, 540–549. 10.1016/j.plaphy.2009.01.004 19230697

[B23] Rolim A. E. H. Henrique-Araujo R. Ferraz E. G. de Araujo Alves Dultra F. K. Fernandez L. G. (2015). Lipidomics in the Study of Lipid Metabolism: Current Perspectives in the Omic Sciences. Gene 554, 131–139. 10.1016/j.gene.2014.10.039 25445283

[B24] Rowland O. Zheng H. Hepworth S. R. Lam P. Jetter R. Kunst L. (2006). *CER4* Encodes an Alcohol-Forming Fatty Acyl-Coenzyme A Reductase Involved in Cuticular Wax Production in Arabidopsis. Plant Physiol. 142, 866–877. 10.1104/pp.106.086785 16980563 PMC1630741

[B25] Samuels L. Kunst L. Jetter R. (2008). Sealing Plant Surfaces: Cuticular Wax Formation by Epidermal Cells. Annu. Rev. Plant Biol. 59, 683–707. 10.1146/annurev.arplant.59.103006.093219 18251711

[B26] Schuett B. S. Millar T. J. (2013). An Investigation of the Likely Role of (O-Acyl) ω-hydroxy Fatty Acids in Meibomian Lipid Films Using (O-Oleyl) ω-hydroxy Palmitic Acid as a Model. Exp. Eye Res. 115, 57–64. 10.1016/j.exer.2013.06.016 23792170

[B27] Shaheenuzzamn M. Liu T. X. Shi S. D. Wu H. Q. Wang Z. H. (2019). Research Advances on Cuticular Waxes Biosynthesis in Crops: a Review. IJAB 21, 911–921. 10.17957/ijab/15.0975

[B28] Tafolla-Arellano J. C. Baez-Sanudo R. Tiznado-Hernández M. E. (2018). The Cuticle as a Key Factor in the Quality of Horticultural Crops. Scientia Horticulturae 232, 145–152. 10.1016/j.scienta.2018.01.005

[B29] Wang M. Wu H. Xu J. Li C. Wang Y. Wang Z. (2017). Five Fatty Acyl-Coenzyme A Reductases Are Involved in the Biosynthesis of Primary Alcohols in *Aegilops Tauschii* Leaves. Front. Plant Sci. 8, 14. 10.3389/fpls.2017.01012 28659955 PMC5466989

[B30] Wang W. Zhang Y. Xu C. Ren J. Liu X. Black K. (2015a). Cucumber *ECERIFERUM1* (*CsCER1*), Which Influences the Cuticle Properties and Drought Tolerance of Cucumber, Plays a Key Role in VLC Alkanes Biosynthesis. Plant Mol. Biol. 87, 219–233. 10.1007/s11103-014-0271-0 25539982

[B31] Wang Y. Mao H. Lv Y. Chen G. Jiang Y. (2021). Comparative Analysis of Total Wax Content, Chemical Composition and crystal Morphology of Cuticular Wax in Korla Pear under Different Relative Humidity of Storage. Food Chem. 339, 128097. 10.1016/j.foodchem.2020.128097 32979715

[B32] Wang Y. Wang J. Chai G. Li C. Hu Y. Chen X. (2015b). Developmental Changes in Composition and Morphology of Cuticular Waxes on Leaves and Spikes of Glossy and Glaucous Wheat (*Triticum aestivum* L.). Plos One 10, e0141239. 10.1371/journal.pone.0141239 26506246 PMC4624236

[B33] Wang Y. Wang M. Sun Y. Wang Y. Li T. Chai G. (2015c). FAR5, a Fatty Acyl-Coenzyme A Reductase, Is Involved in Primary Alcohol Biosynthesis of the Leaf Blade Cuticular Wax in Wheat (*Triticum aestivum* L.). J. Exp. Bot. 66, 1165–1178. 10.1093/jxb/eru457 25468933 PMC4438443

[B34] Yeats T. H. Buda G. J. Wang Z. Chehanovsky N. Moyle L. C. Jetter R. (2012). The Fruit Cuticles of Wild Tomato Species Exhibit Architectural and Chemical Diversity, Providing a New Model for Studying the Evolution of Cuticle Function. Plant J. 69, 655–666. 10.1111/j.1365-313X.2011.04820.x 22007785 PMC3736592

[B35] Zhang J.-Y. Broeckling C. D. Blancaflor E. B. Sledge M. K. Sumner L. W. Wang Z.-Y. (2005). Overexpression of *WXP1*, a Putative *Medicago Truncatula* AP2 Domain-Containing Transcription Factor Gene, Increases Cuticular Wax Accumulation and Enhances Drought Tolerance in Transgenic Alfalfa (*Medicago Sativa*). Plant J. 42, 689–707. 10.1111/j.1365-313X.2005.02405.x 15918883

[B36] Zhang Z. Wang W. Li W. (2013). Genetic Interactions Underlying the Biosynthesis and Inhibition of β-Diketones in Wheat and Their Impact on Glaucousness and Cuticle Permeability. Plos One 8, e54129. 10.1371/journal.pone.0054129 23349804 PMC3547958

[B37] Zheng X. Liu C. Qiao L. Zhao J. Han R. Wang X. (2020). The MYB Transcription Factor *TaPHR3-A1* Is Involved in Phosphate Signaling and Governs Yield-Related Traits in Bread Wheat. J. Exp. Bot. 71, 5808–5822. 10.1093/jxb/eraa355 32725154

[B38] Zhou X. Jenks M. A. Liu J. Liu A. Zhang X. Xiang J. (2014). Overexpression of Transcription Factor *OsWR2* Regulates Wax and Cutin Biosynthesis in rice and Enhances its Tolerance to Water Deficit. Plant Mol. Biol. Rep. 32, 719–731. 10.1007/s11105-013-0687-8

