# Supplementary material for: Analysis of Wheat Wax Regulation Mechanism by Liposome and Transcriptome
Source: Front Genet. 2021 Dec 6;12:757920. doi: 10.3389/fgene.2021.757920 (PMC8687455; doi:10.3389/fgene.2021.757920)
Supplement: Supplementary file 1 [file DataSheet4.docx]

## Supplementary Material

**Supplementary Figures and Tables**

**Supplementary Figure 1.** Differential gene GO enrichment map

**Supplementary Figure 2.** qRT-PCR validation of random DEGs

**Supplementary Table 1.** List of primers used for qRT-PCR

**Supplementary Table 2.** The significant differences in lipid ion between the two mutants


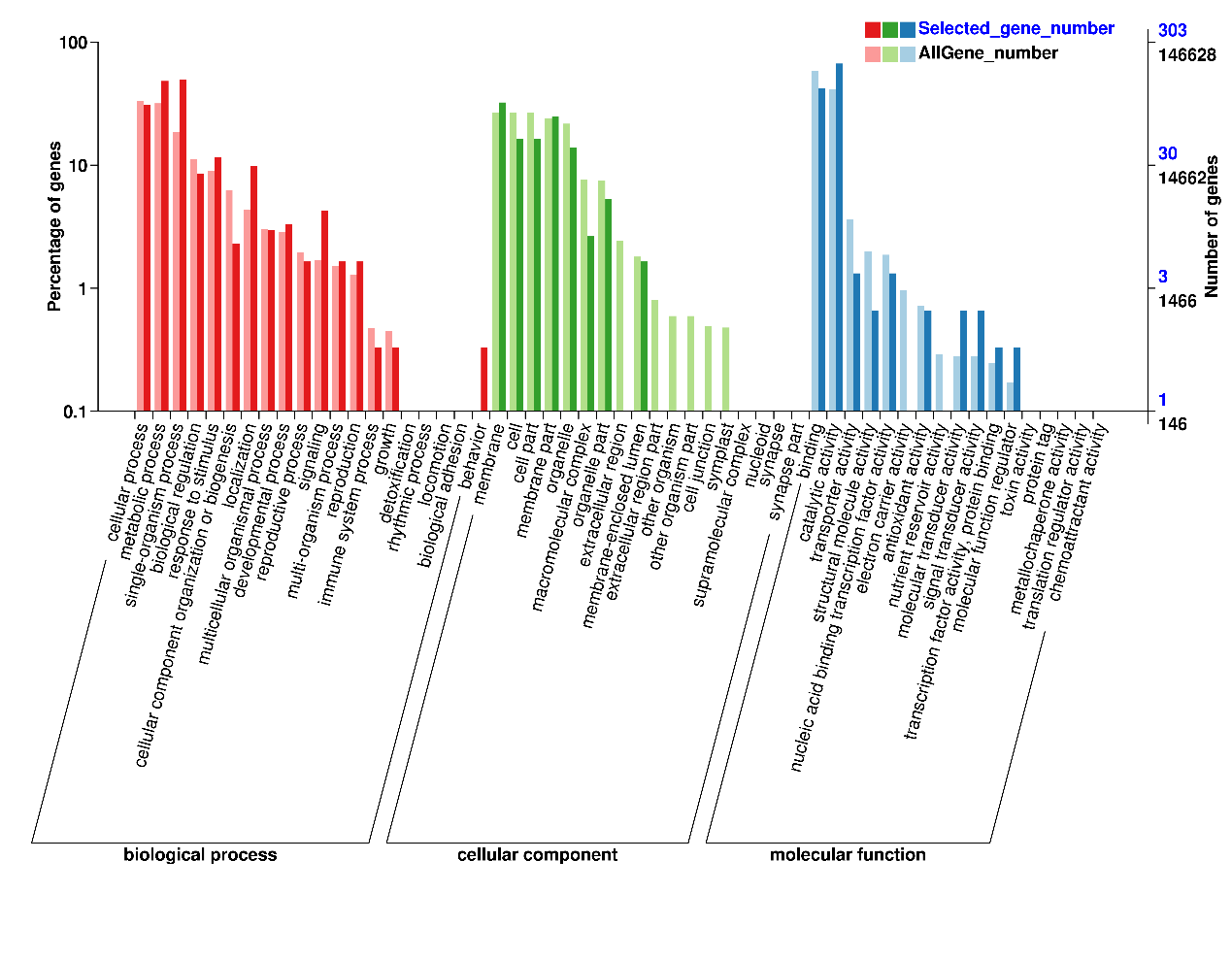


**Supplementary Figure 1.** Differential gene GO enrichment map


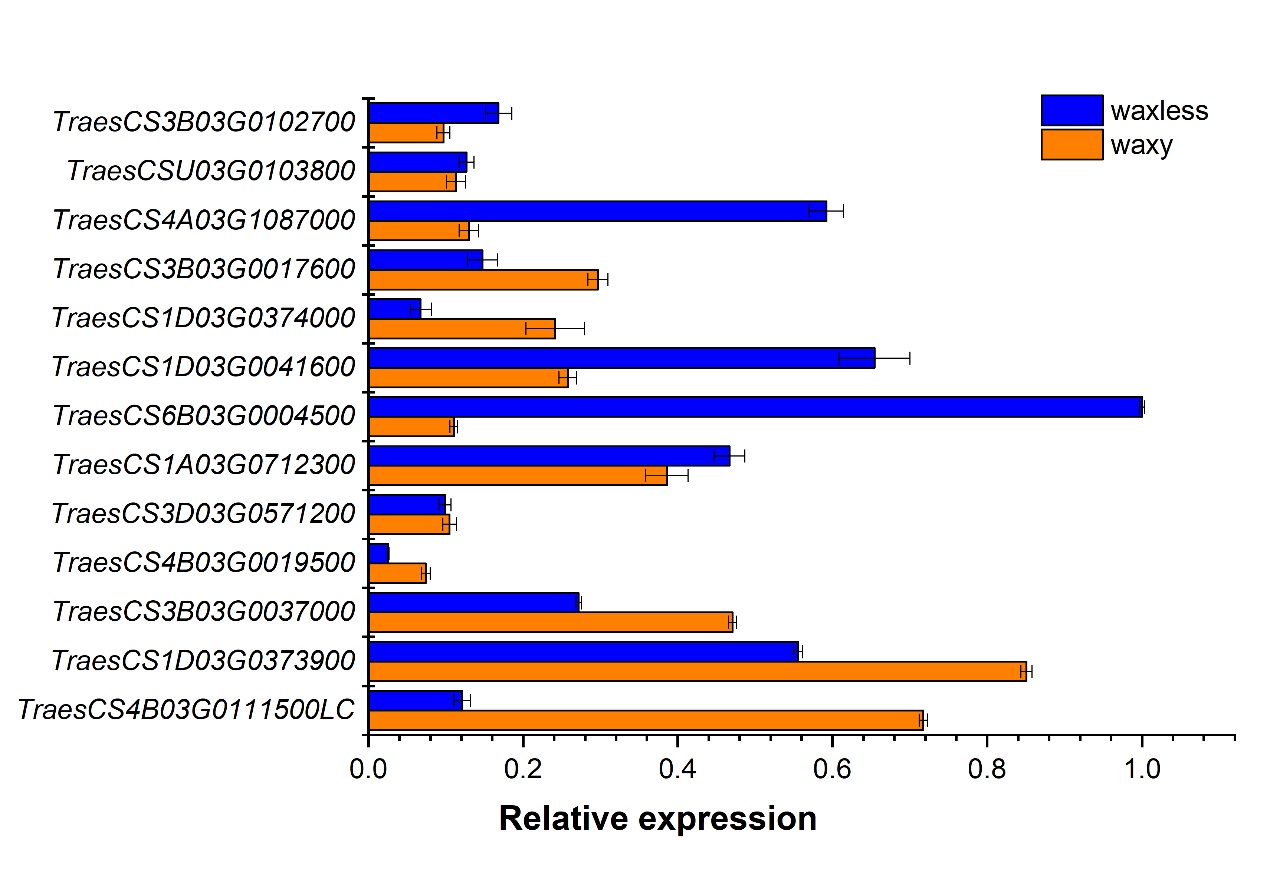
 **Supplementary Figure 2.** qRT-PCR validation of random DEGs

**Supplementary Table 1.** List of primers used for qRT-PCR

| Gene ID | Sense | Antisense |
| --- | --- | --- |
| *TraesCS6B03G0004500* | CCGTGAAGGTGATTGACA | TTGGCACTGAGGTTCTTG |
| *TraesCS1D03G0041600* | TAGCATCATACTCAGCACTT | ATTGTAGCCACGGTTCAG |
| *TraesCS1A03G0712300* | GAGCAAGAATACCGTCAGT | TGGAGCGTGGATAAGGAA |
| *TraesCS3D03G0571200* | TGGAGGAGGAATGCGTAT | GACTGCTGTGGTGATGTT |
| *TraesCS4B03G0111500LC* | AACTCTCCTAATACCGCAACCT | TGCTGGAACCCGTGAACTG |
| *TraesCS1D03G0373900* | GGCATACGGTTCTTGGTT | CCTCCTTCTCCTTGTTCAG |
| *TraesCS3B03G0037000* | GAGACGGAAGGTGCTGACA | GTGGACAACATCGCTACATAGT |
| *TraesCS4B03G0019500* | TTCTTCCTCCTCCGTCTC | GCTTCTTCTCTGGCTCTG |
| *TraesCS1D03G0374000* | CTCCACCACACTCAGTTC | CTTCACATATTGCCTTCACA |
| *TraesCS3B03G0017600* | GAGGACTATGTAGCGATGTT | GGTCTTGATGAGCGTGATA |
| *TraesCS4A03G1087000* | CTGAGCACAAGGACTATGG | TAACGATAACGACGCAATTC |
| *TraesCSU03G0103800* | GCAAGCACATCTCGGTAG | AGGAAGGAAGGACACATACT |
| *TraesCS3B03G0102700* | CGTAGTGAAGCGTGAAGAT | CCAAGTAGCAGGCAAGTC |

**Supplementary Table 2.** The significant differences in lipid ion between the two mutants

| Class | LipidIon | IonFormula | Calmz | RT-(min) | VIP | FC（wxless/waxy） | PValue |
| --- | --- | --- | --- | --- | --- | --- | --- |
| WE | WE(28:1_22:0)+H | H99 C50 O2 | 731.764 | 21.557 | 1.037 | 0.026 | 2.19E-06 |
| WE | WE(28:1_20:0)+H | H95 C48 O2 | 703.733 | 20.667 | 1.038 | 0.0594 | 1.68E-09 |
| WE | WE(28:0_21:3)+H | H93 C49 O2 | 713.717 | 17.756 | 1.038 | 0.0919 | 3.21E-06 |
| WE | WE(28:1_18:0)+H | H91 C46 O2 | 675.701 | 19.485 | 1.039 | 0.0299 | 6.09E-07 |
| WE | WE(26:0_21:3)+H | H89 C47 O2 | 685.686 | 16.346 | 1.039 | 0.0322 | 1.13E-06 |
| WE | WE(28:1_16:0)+H | H87 C44 O2 | 647.67 | 18.216 | 1.038 | 0.0509 | 1.41E-05 |
| WE | WE(28:1_14:0)+H | H83 C42 O2 | 619.639 | 16.943 | 1.037 | 0.0698 | 5.44E-05 |
| WE | WE(24:0_14:1)+H | H75 C38 O2 | 563.576 | 15.814 | 1.039 | 0.0231 | 3.44E-07 |
| WE | WE(15:1_22:0)+H | H73 C37 O2 | 549.561 | 14.501 | 1.038 | 0.1324 | 1.49E-05 |
| WE | WE(15:1_18:0)+H | H65 C33 O2 | 493.498 | 12.355 | 1.034 | 0.2369 | 1.65E-04 |
| WE | WE(13:0_20:2)+H | H63 C33 O2 | 491.482 | 11.123 | 1.038 | 0.1252 | 9.43E-06 |
| WE | WE(16:0_17:3)+H | H61 C33 O2 | 489.467 | 7.275 | 1.038 | 0.1072 | 1.98E-05 |
| WE | WE(13:0_19:2)+H | H61 C32 O2 | 477.467 | 10.81 | 1.035 | 0.099 | 1.03E-04 |
| WE | WE(19:1_12:0)+H | H61 C31 O2 | 465.467 | 9.232 | 1.034 | 0.0933 | 1.40E-04 |
| WE | WE(12:0_19:3)+NH4 | H60 C31 O2 N1 | 478.462 | 6.817 | 1.037 | 0.0425 | 4.35E-05 |
| WE | WE(12:0_19:3)+NH4 | H60 C31 O2 N1 | 478.462 | 7.269 | 1.039 | 0.0434 | 3.09E-07 |
| WE | WE(17:1_14:2)+NH4 | H60 C31 O2 N1 | 478.462 | 9.648 | 1.038 | 0.0791 | 1.58E-05 |
| WE | WE(15:0_16:2)+H | H59 C31 O2 | 463.451 | 10.343 | 1.037 | 0.043 | 1.03E-06 |
| WE | WE(16:0_15:2)+H | H59 C31 O2 | 463.451 | 9.128 | 1.038 | 0.1351 | 3.13E-05 |
| WE | WE(13:0_18:2)+H | H59 C31 O2 | 463.451 | 19.86 | 1.034 | 0.1819 | 1.95E-04 |
| WE | WE(12:0_19:4)+NH4 | H58 C31 O2 N1 | 476.446 | 6.755 | 1.036 | 0.1761 | 1.05E-05 |
| WE | WE(15:1_16:2)+H | H57 C31 O2 | 461.435 | 8.696 | 1.037 | 0.0448 | 1.65E-06 |
| WE | WE(13:0_18:3)+H | H57 C31 O2 | 461.435 | 6.311 | 1.034 | 0.0677 | 1.49E-04 |
| WE | WE(16:1_15:2)+H | H57 C31 O2 | 461.435 | 10.299 | 1.036 | 0.1197 | 7.32E-05 |
| WE | WE(13:0_17:2)+H | H57 C30 O2 | 449.435 | 9.718 | 1.035 | 0.126 | 1.44E-04 |
| WE | WE(8:0_21:3)+NH4 | H56 C29 O2 N1 | 450.431 | 8.75 | 1.039 | 0.0498 | 1.08E-06 |
| WE | WE(11:0_18:2)+H | H55 C29 O2 | 435.42 | 9.451 | 1.037 | 0.1567 | 7.85E-06 |
| WE | WE(8:0_21:3)+H | H53 C29 O2 | 433.404 | 9.176 | 1.037 | 0.064 | 3.99E-05 |
| WE | WE(3:0_20:3)+H | H41 C23 O2 | 349.31 | 6.76 | 1.038 | 0.0581 | 3.71E-06 |
| WE | WE(19:1)-H | H35 C19 O2 | 295.264 | 8.639 | 1.038 | 0.0802 | 5.09E-06 |
| TG | TG(70:0)+NH4 | C73 H146 O6 N1 | 1133.114 | 25.003 | 1.035 | 0.1472 | 1.45E-04 |
| TG | TG(28:0_16:0_22:0)+NH4 | C69 H138 O6 N1 | 1077.052 | 25.25 | 1.038 | 0.1262 | 1.91E-05 |
| CL | CL(56:2)-2H | C65 H120 O17 P2 | 617.401 | 7.945 | 1.037 | 0.2104 | 3.56E-05 |
| TG | TG(18:0e_19:0_24:2)+H | C64 H123 O5 | 971.937 | 23.904 | 1.038 | 0.2477 | 1.01E-06 |
| TG | TG(25:0_18:2_18:2)+H | C64 H117 O6 | 981.884 | 20.945 | 1.037 | 0.0127 | 3.04E-05 |
| TG | TG(19:0_19:0_21:1)+NH4 | C62 H122 O6 N1 | 976.927 | 21.332 | 1.038 | 0.0897 | 5.36E-06 |
| TG | TG(20:2e_16:0_23:0)+H | C62 H119 O5 | 943.905 | 23.252 | 1.038 | 0.1299 | 1.80E-07 |
| TG | TG(20:3e_16:0_23:0)+H | C62 H117 O5 | 941.89 | 22.013 | 1.038 | 0.0309 | 1.58E-06 |
| TG | TG(20:3e_18:2_20:2)+H | C61 H107 O5 | 919.811 | 21.622 | 1.037 | 0.249 | 3.52E-05 |
| TG | TG(12:1e_20:0_22:0)+NH4 | C57 H114 O5 N1 | 892.869 | 21.634 | 1.037 | 0.132 | 3.63E-05 |
| TG | TG(20:0e_16:0_16:0)+NH4 | C55 H112 O5 N1 | 866.854 | 21.027 | 1.039 | 0.0333 | 3.78E-06 |
| TG | TG(18:3e_16:0_18:0)+H | C55 H103 O5 | 843.78 | 19.589 | 1.036 | 0.0066 | 7.24E-05 |
| TG | TG(50:3e)+H | C53 H99 O5 | 815.749 | 21.883 | 1.038 | 0.0692 | 1.11E-07 |
| TG | TG(12:1e_18:2_20:5)+Na | C53 H88 O5 Na1 | 827.652 | 21.062 | 1.035 | 0.195 | 1.40E-04 |
| DG | DG(28:0_22:0)+NH4 | C53 H108 O5 N1 | 838.822 | 22.534 | 1.033 | 0.1957 | 2.61E-04 |
| DG | DG(34:1_16:0)+NH4 | C53 H106 O5 N1 | 836.807 | 16.183 | 1.038 | 0.0325 | 2.97E-07 |
| OAHFA | OAHFA(52:2)-H | C52 H97 O4 | 785.739 | 22.396 | 1.038 | 0.0659 | 1.25E-05 |
| OAHFA | OAHFA(51:1)-H | C51 H97 O4 | 773.739 | 22.958 | 1.038 | 0.2262 | 2.45E-05 |
| TG | TG(12:1e_18:0_18:3)+H | C51 H93 O5 | 785.702 | 15.764 | 1.038 | 0.0227 | 6.91E-07 |
| TG | TG(12:1e_18:0_18:3)+H | C51 H93 O5 | 785.702 | 17.405 | 1.037 | 0.2156 | 2.26E-05 |
| DG | DG(32:0_16:0)+NH4 | C51 H104 O5 N1 | 810.791 | 18.368 | 1.036 | 0.1476 | 9.01E-05 |
| TG | TG(12:1e_16:0_20:0)+NH4 | C51 H102 O5 N1 | 808.775 | 18.422 | 1.039 | 0.0233 | 1.32E-06 |
| TG | TG(12:0e_16:0_20:0)+Na | C51 H100 O5 Na1 | 815.746 | 18.366 | 1.033 | 0.1005 | 1.72E-04 |
| OAHFA | OAHFA(50:2)-H | C50 H93 O4 | 757.708 | 21.662 | 1.038 | 0.0567 | 4.61E-07 |
| TG | TG(18:0_12:2_17:1)+H | C50 H91 O6 | 787.681 | 14.334 | 1.038 | 0.0151 | 9.04E-06 |
| TG | TG(12:0_17:1_18:2)+H | C50 H91 O6 | 787.681 | 13.837 | 1.038 | 0.0255 | 1.11E-08 |
| TG | TG(20:4e_13:0_14:1)+H | C50 H89 O5 | 769.67 | 15.279 | 1.039 | 0.0441 | 7.16E-07 |
| TG | TG(20:4e_13:0_14:2)+H | C50 H87 O5 | 767.655 | 15.463 | 1.033 | 0.0628 | 1.55E-04 |
| TG | TG(12:0e_16:0_18:4)+H | C49 H89 O5 | 757.67 | 16.761 | 1.037 | 0.0058 | 3.00E-05 |
| TG | TG(20:1e_10:3_16:0)+H | C49 H89 O5 | 757.67 | 17.484 | 1.038 | 0.1072 | 1.08E-06 |
| Cer | Cer(d48:1)+H | C48 H96 O3 N1 | 734.738 | 21.824 | 1.036 | 0.1507 | 6.70E-05 |
| DG | DG(45:2)+H | C48 H91 O5 | 747.686 | 15.278 | 1.039 | 0 | 6.49E-07 |
| TG | TG(14:1e_14:1_17:0)+H | C48 H91 O5 | 747.686 | 14.332 | 1.038 | 0.0602 | 4.44E-07 |
| Cer | Cer(m48:4)+H | C48 H90 O2 N1 | 712.697 | 16.97 | 1.036 | 0.1107 | 4.72E-05 |
| TG | TG(20:3e_9:0_16:0)+H | C48 H89 O5 | 745.67 | 15.455 | 1.038 | 0.0741 | 2.68E-05 |
| OAHFA | OAHFA(48:2)-H | C48 H89 O4 | 729.677 | 20.806 | 1.038 | 0.0941 | 2.18E-06 |
| TG | TG(15:0_14:3_16:0)+H | C48 H87 O6 | 759.65 | 12.775 | 1.038 | 0.0895 | 1.19E-06 |
| TG | TG(16:1e_12:2_17:1)+H | C48 H87 O5 | 743.655 | 13.602 | 1.039 | 0.003 | 6.03E-06 |
| TG | TG(11:0_14:1_20:3)+H | C48 H85 O6 | 757.634 | 11.099 | 1.038 | 0.0284 | 2.22E-06 |
| TG | TG(20:0e_11:4_14:1)+H | C48 H85 O5 | 741.639 | 13.923 | 1.038 | 0.0192 | 1.06E-08 |
| TG | TG(20:0e_11:4_14:1)+H | C48 H85 O5 | 741.639 | 14.392 | 1.037 | 0.0546 | 5.18E-05 |
| TG | TG(20:0e_11:4_14:2)+H | C48 H83 O5 | 739.624 | 14.24 | 1.038 | 0.0963 | 6.44E-06 |
| TG | TG(24:1_10:4_11:2)+Na | C48 H78 O6 Na1 | 773.569 | 13.752 | 1.037 | 0.1839 | 5.87E-06 |
| DG | DG(32:1_12:0)+NH4 | C47 H94 O5 N1 | 752.713 | 14.379 | 1.036 | 0.0222 | 5.79E-05 |
| TG | TG(14:0e_14:1_16:0)+NH4 | C47 H94 O5 N1 | 752.713 | 17.519 | 1.038 | 0.0224 | 2.21E-05 |
| DG | DG(44:2e)+NH4 | C47 H94 O4 N1 | 736.718 | 19.814 | 1.037 | 0.1536 | 5.10E-05 |
| TG | TG(12:0e_16:0_16:0)+Na | C47 H92 O5 Na1 | 759.684 | 19.988 | 1.037 | 0.1951 | 6.19E-07 |
| Cer | Cer(d21:0_26:2)+H | C47 H92 O3 N1 | 718.707 | 17.151 | 1.036 | 0.0974 | 6.61E-05 |
| OAHFA | OAHFA(16:0_31:0)-H | C47 H91 O4 | 719.692 | 21.834 | 1.034 | 0.1167 | 1.66E-04 |
| DG | DG(44:3e)+H | C47 H89 O4 | 717.676 | 16.783 | 1.035 | 0.0014 | 7.66E-05 |
| OAHFA | OAHFA(16:0_31:1)-H | C47 H89 O4 | 717.677 | 16.781 | 1.036 | 0.0158 | 5.94E-06 |
| TG | TG(12:1e_16:1_16:1)+H | C47 H87 O5 | 731.655 | 11.318 | 1.038 | 0 | 7.18E-07 |
| DG | DG(44:4e)+H | C47 H87 O4 | 715.66 | 19.812 | 1.037 | 0.1098 | 4.78E-05 |
| TG | TG(17:0_10:2_17:1)+H | C47 H85 O6 | 745.634 | 14.327 | 1.038 | 0.0959 | 2.28E-06 |
| Cer | Cer(m46:0)+H | C46 H94 O2 N1 | 692.728 | 15.058 | 1.039 | 0.0302 | 4.07E-06 |
| TG | TG(15:0_12:0_16:0)+NH4 | C46 H92 O6 N1 | 754.692 | 16.32 | 1.037 | 0.1387 | 3.28E-05 |
| Cer | Cer(d46:0+O)+H-H2O | C46 H92 O3 N1 | 706.707 | 20.881 | 1.034 | 0.1498 | 1.75E-04 |
| TG | TG(16:0e_11:2_16:0)+H | C46 H87 O5 | 719.655 | 14.009 | 1.037 | 0.0033 | 6.09E-05 |
| ChE | ChE(19:0)+NH4 | C46 H86 O2 N1 | 684.665 | 15.942 | 1.036 | 0.0296 | 5.38E-05 |
| TG | TG(16:0e_11:3_16:0)+H | C46 H85 O5 | 717.639 | 11.087 | 1.039 | 0 | 1.01E-05 |
| TG | TG(16:1e_12:2_15:0)+H | C46 H85 O5 | 717.639 | 14.286 | 1.038 | 0.0695 | 2.74E-07 |
| DG | DG(43:4e)+H | C46 H85 O4 | 701.644 | 14.136 | 1.034 | 0.152 | 1.46E-04 |
| OAHFA | OAHFA(46:2)-H | C46 H85 O4 | 701.645 | 19.593 | 1.03 | 0.2271 | 3.26E-04 |
| TG | TG(18:3e_11:0_14:4)+NH4 | C46 H80 O5 N1 | 726.603 | 12.081 | 1.038 | 0.1581 | 1.19E-05 |
| DG | DG(42:1e)+NH4 | C45 H92 O4 N1 | 710.702 | 19.808 | 1.038 | 0.1552 | 9.34E-07 |
| TG | TG(16:0_10:0_16:0)+NH4 | C45 H90 O6 N1 | 740.676 | 14.354 | 1.036 | 0.111 | 7.35E-05 |
| DG | DG(42:2e)+NH4 | C45 H90 O4 N1 | 708.686 | 18.45 | 1.035 | 0.1717 | 1.02E-04 |
| OAHFA | OAHFA(16:0_29:0)-H | C45 H87 O4 | 691.661 | 20.962 | 1.036 | 0.1289 | 9.36E-05 |
| TG | TG(12:0e_10:0_20:3)+H | C45 H83 O5 | 703.624 | 17.448 | 1.036 | 0.1871 | 9.58E-06 |
| Cer | Cer(m44:0)+H | C44 H90 O2 N1 | 664.697 | 13.932 | 1.037 | 0.0954 | 4.18E-05 |
| Cer | Cer(d44:4)+H | C44 H82 O3 N1 | 672.629 | 11.306 | 1.036 | 0.0618 | 7.73E-05 |
| DG | DG(40:1e)+NH4 | C43 H88 O4 N1 | 682.671 | 18.482 | 1.032 | 0.1183 | 1.98E-04 |
| Cer | Cer(m19:0_24:0+O)+H | C43 H88 O3 N1 | 666.676 | 14.559 | 1.039 | 0.077 | 6.97E-07 |
| Cer | Cer(m42:0)+H | C42 H86 O2 N1 | 636.665 | 12.894 | 1.036 | 0.1234 | 9.89E-05 |
| Cer | Cer(d23:0_18:0)+H | C41 H84 O3 N1 | 638.645 | 13.164 | 1.038 | 0.0587 | 3.15E-07 |
| Cer | Cer(d23:0_17:1)+H | C40 H80 O3 N1 | 622.613 | 17.004 | 1.037 | 0.1578 | 4.73E-05 |
| DG | DG(37:4e)+H | C40 H73 O4 | 617.55 | 15.722 | 1.038 | 0 | 3.13E-05 |
| DG | DG(36:1e)+NH4 | C39 H80 O4 N1 | 626.608 | 16.026 | 1.038 | 0.1067 | 3.46E-06 |
| Cer | Cer(d16:1_22:3)+H | C38 H70 O3 N1 | 588.535 | 8.524 | 1.038 | 0.065 | 1.07E-08 |
| DG | DG(35:2D7)+H | C38 H64 O5 D7 | 614.574 | 7.71 | 1.038 | 0.0226 | 2.79E-05 |
| DG | DG(34:1e)+NH4 | C37 H76 O4 N1 | 598.577 | 6.326 | 1.037 | 0.0057 | 2.47E-06 |
| Cer | Cer(d21:0_16:0)+H | C37 H76 O3 N1 | 582.582 | 10.138 | 1.037 | 0.2171 | 2.93E-05 |
| DG | DG(18:2e_16:0)+NH4 | C37 H74 O4 N1 | 596.561 | 11.159 | 1.034 | 0.106 | 1.39E-04 |
| DG | DG(33:1e)+NH4 | C36 H74 O4 N1 | 584.561 | 14.199 | 1.038 | 0.0278 | 1.04E-05 |
| Cer | Cer(t36:4)+H | C36 H66 O4 N1 | 576.499 | 6.71 | 1.038 | 0.0917 | 2.10E-07 |
| OAHFA | OAHFA(36:2)-H | C36 H65 O4 | 561.489 | 13.129 | 1.037 | 0.1841 | 3.27E-06 |
| MG | MG(32:1)+NH4 | C35 H72 O4 N1 | 570.546 | 6.394 | 1.038 | 0.0216 | 3.40E-05 |
| MG | MG(32:1)+NH4 | C35 H72 O4 N1 | 570.546 | 8.707 | 1.035 | 0.0345 | 9.77E-05 |
| DG | DG(32:1e)+NH4 | C35 H72 O4 N1 | 570.546 | 13.501 | 1.038 | 0.0534 | 1.15E-05 |
| Cer | Cer(d35:0)+H | C35 H72 O3 N1 | 554.551 | 9.437 | 1.039 | 0.0624 | 5.55E-06 |
| Cer | Cer(d17:0_18:0)+H | C35 H72 O3 N1 | 554.551 | 10.032 | 1.038 | 0.2313 | 9.96E-07 |
| Cer | Cer(d17:0_18:0)+H | C35 H72 O3 N1 | 554.551 | 11.273 | 1.037 | 0.2494 | 5.57E-05 |
| Cer | Cer(m35:0)+H-H2O | C35 H70 O1 N1 | 520.545 | 16.772 | 1.035 | 0.2385 | 1.37E-04 |
| DG | DG(31:1e)+NH4 | C34 H70 O4 N1 | 556.53 | 13.188 | 1.037 | 0.0229 | 4.06E-05 |
| Cer | Cer(d18:0_16:0)+H | C34 H70 O3 N1 | 540.535 | 11.124 | 1.035 | 0.1373 | 1.06E-04 |
| TG | TG(4:0_13:0_14:2)+Na | C34 H60 O6 Na1 | 587.428 | 8.643 | 1.039 | 0.0357 | 1.24E-06 |
| Cer | Cer(d17:0_16:0)+H | C33 H68 O3 N1 | 526.519 | 10.305 | 1.039 | 0.1934 | 2.03E-06 |
| Cer | Cer(t17:1_16:0)+H | C33 H66 O4 N1 | 540.499 | 11.376 | 1.037 | 0.2441 | 2.24E-05 |
| Cer | Cer(m19:1_14:0+O)+H | C33 H66 O3 N1 | 524.504 | 10.354 | 1.038 | 0.0942 | 7.67E-06 |
| Cer | Cer(d17:1_16:0)+H | C33 H66 O3 N1 | 524.504 | 10.322 | 1.037 | 0.1015 | 4.20E-06 |
| Cer | Cer(m17:0_16:1)+H | C33 H66 O2 N1 | 508.509 | 10.646 | 1.036 | 0.0658 | 8.69E-05 |
| Cer | Cer(m33:0)+H-H2O | C33 H66 O1 N1 | 492.514 | 16.081 | 1.037 | 0.219 | 1.59E-05 |
| DG | DG(30:1e)+H | C33 H65 O4 | 525.488 | 8.165 | 1.037 | 0.1151 | 2.87E-06 |
| DG | DG(30:4e)+H | C33 H59 O4 | 519.441 | 8.307 | 1.035 | 0.0863 | 1.27E-04 |
| DG | DG(30:4e)+H | C33 H59 O4 | 519.441 | 11.337 | 1.035 | 0.1713 | 9.95E-05 |
| DG | DG(30:5e)+H | C33 H57 O4 | 517.425 | 6.411 | 1.038 | 0.0263 | 1.27E-05 |
| Cer | Cer(t32:1+O)+NH4 | C32 H67 O5 N2 | 559.504 | 13.318 | 1.038 | 0.0429 | 1.30E-05 |
| Cer | Cer(d18:2_14:0+O)+NH4 | C32 H65 O4 N2 | 541.494 | 5.948 | 1.037 | 0.0672 | 3.56E-05 |
| Cer | Cer(t32:1+O)-H | C32 H62 O5 N1 | 540.463 | 5.816 | 1.038 | 0.1051 | 8.40E-06 |
| OAHFA | OAHFA(32:1)-H | C32 H59 O4 | 507.442 | 10.674 | 1.036 | 0.0808 | 5.82E-05 |
| DG | DG(29:4e)+H | C32 H57 O4 | 505.425 | 8.246 | 1.038 | 0.0874 | 2.12E-05 |
| DG | DG(29:4e)+H | C32 H57 O4 | 505.425 | 7.817 | 1.038 | 0.1256 | 1.75E-05 |
| DG | DG(28:1e)+NH4 | C31 H64 O4 N1 | 514.483 | 9.117 | 1.039 | 0.0158 | 5.10E-07 |
| DG | DG(28:1e)+NH4 | C31 H64 O4 N1 | 514.483 | 8.144 | 1.038 | 0.0232 | 1.64E-05 |
| Cer | Cer(t16:0_15:0)+H | C31 H64 O4 N1 | 514.483 | 10.272 | 1.039 | 0.1291 | 7.28E-06 |
| Cer | Cer(m17:0_14:0)+H | C31 H64 O2 N1 | 482.493 | 14.784 | 1.038 | 0.2177 | 7.20E-06 |
| DG | DG(28:0e)+H | C31 H63 O4 | 499.472 | 5.151 | 1.039 | 0 | 9.09E-07 |
| DG | DG(14:0e_14:2)+NH4 | C31 H62 O4 N1 | 512.467 | 6.382 | 1.037 | 0.0555 | 4.57E-06 |
| Cer | Cer(d16:1_15:0)+H | C31 H62 O3 N1 | 496.472 | 6.224 | 1.037 | 0.0152 | 3.80E-05 |
| Cer | Cer(d19:1_12:0)+H | C31 H62 O3 N1 | 496.472 | 8.162 | 1.036 | 0.0204 | 9.54E-05 |
| Cer | Cer(d15:0_16:1)+H | C31 H62 O3 N1 | 496.472 | 5.816 | 1.037 | 0.0237 | 4.22E-05 |
| Cer | Cer(d15:1_16:0)+H | C31 H62 O3 N1 | 496.472 | 8.597 | 1.039 | 0.0479 | 1.81E-06 |
| Cer | Cer(d16:0_15:1)+H | C31 H62 O3 N1 | 496.472 | 10.332 | 1.038 | 0.0587 | 6.17E-06 |
| Cer | Cer(d16:1_15:0)+H | C31 H62 O3 N1 | 496.472 | 6.689 | 1.036 | 0.0813 | 6.80E-05 |
| Cer | Cer(m17:1_14:0+O)+H | C31 H62 O3 N1 | 496.472 | 7.646 | 1.038 | 0.0873 | 7.37E-07 |
| DG | DG(28:1e)+H | C31 H61 O4 | 497.456 | 10.263 | 1.037 | 0.0078 | 2.91E-06 |
| MG | MG(28:1)+H | C31 H61 O4 | 497.456 | 6.322 | 1.038 | 0.0228 | 2.61E-06 |
| Cer | Cer(d15:2_16:0)+H | C31 H60 O3 N1 | 494.457 | 8.128 | 1.038 | 0.0442 | 2.90E-09 |
| Cer | Cer(d14:2_17:0)+H | C31 H60 O3 N1 | 494.457 | 5.767 | 1.037 | 0.1562 | 9.79E-06 |
| OAHFA | OAHFA(31:0)-H | C31 H59 O4 | 495.442 | 6.295 | 1.039 | 0.02 | 8.36E-06 |
| OAHFA | OAHFA(15:0_16:0)-H | C31 H59 O4 | 495.442 | 8.63 | 1.038 | 0.0212 | 3.05E-08 |
| OAHFA | OAHFA(16:0_15:0)-H | C31 H59 O4 | 495.442 | 14.907 | 1.038 | 0.0525 | 6.52E-07 |
| OAHFA | OAHFA(16:0_15:0)-H | C31 H59 O4 | 495.442 | 13.286 | 1.036 | 0.092 | 5.49E-05 |
| OAHFA | OAHFA(17:0_14:0)-H | C31 H59 O4 | 495.442 | 14.068 | 1.038 | 0.0976 | 1.42E-05 |
| Cer | Cer(d31:2)+H-H2O | C31 H58 O2 N1 | 476.446 | 11.129 | 1.037 | 0.0396 | 4.54E-05 |
| Cer | Cer(d31:2)+H-H2O | C31 H58 O2 N1 | 476.446 | 6.762 | 1.038 | 0.0551 | 2.54E-07 |
| OAHFA | OAHFA(31:1)-H | C31 H57 O4 | 493.426 | 21.881 | 1.037 | 0.0281 | 1.51E-06 |
| OAHFA | OAHFA(31:1)-H | C31 H57 O4 | 493.426 | 9.229 | 1.039 | 0.0747 | 8.53E-07 |
| OAHFA | OAHFA(16:0_15:1)-H | C31 H57 O4 | 493.426 | 10.186 | 1.037 | 0.1051 | 2.95E-06 |
| OAHFA | OAHFA(15:0_16:1)-H | C31 H57 O4 | 493.426 | 23.57 | 1.037 | 0.2073 | 4.95E-05 |
| DG | DG(27:1)+NH4 | C30 H60 O5 N1 | 514.447 | 8.405 | 1.037 | 0.0578 | 5.39E-05 |
| OAHFA | OAHFA(30:1)-H | C30 H55 O4 | 479.411 | 10.509 | 1.037 | 0.0166 | 5.45E-05 |
| TG | TG(8:0_8:0_10:0)+NH4 | C29 H58 O6 N1 | 516.426 | 8.637 | 1.038 | 0.1378 | 4.61E-06 |
| Cer | Cer(d14:1_15:0)+H | C29 H58 O3 N1 | 468.441 | 9.18 | 1.037 | 0.1011 | 3.63E-05 |
| Cer | Cer(m17:1_12:0)+H | C29 H58 O2 N1 | 452.446 | 8.725 | 1.038 | 0.0187 | 2.45E-05 |
| TG | TG(6:0_8:0_12:1)+H | C29 H53 O6 | 497.384 | 8.915 | 1.035 | 0.1582 | 1.18E-04 |
| DG | DG(16:2e_8:0)+NH4 | C27 H54 O4 N1 | 456.405 | 8.582 | 1.037 | 0.1789 | 3.02E-05 |
